# Simulation-Based Estimation of SARS-CoV-2 Infections Associated With School Closures and Community-Based Nonpharmaceutical Interventions in Ontario, Canada

**DOI:** 10.1001/jamanetworkopen.2021.3793

**Published:** 2021-03-31

**Authors:** David Naimark, Sharmistha Mishra, Kali Barrett, Yasin A. Khan, Stephen Mac, Raphael Ximenes, Beate Sander

**Affiliations:** 1 Institute of Health Policy, Management and Evaluation, University of Toronto, Toronto, Canada; 2 Toronto Health Economics and Technology Assessment Collaborative, University Health Network, Toronto, Canada; 3 Sunnybrook Health Sciences Centre, Toronto, Canada; 4 Department of Medicine, University of Toronto, Toronto, Canada; 5 Dalla Lana School of Public Health, University of Toronto, Toronto, Canada; 6 Institute of Medical Sciences, University of Toronto, Toronto, Canada; 7 MAP Centre for Urban Health Solutions, Li Ka Shing Knowledge Institute, St Michael’s Hospital, Toronto, Canada; 8 University Health Network, Toronto, Canada; 9 Escola de Matemática Aplicada, Fundação Getúlio Vargas, Rio de Janeiro, Brasil; 10 ICES, Toronto, Canada; 11 Public Health Ontario, Toronto, Canada

## Abstract

**Importance:**

Resurgent COVID-19 cases have resulted in the reinstitution of nonpharmaceutical interventions, including school closures, which can have adverse effects on families. Understanding the associations of school closures with the number of incident and cumulative COVID-19 cases is critical for decision-making.

**Objective:**

To estimate the association of schools being open or closed with the number of COVID-19 cases compared with community-based nonpharmaceutical interventions.

**Design, Setting, and Participants:**

This decision analytical modelling study developed an agent-based transmission model using a synthetic population of 1 000 000 individuals based on the characteristics of the population of Ontario, Canada. Members of the synthetic population were clustered into households, neighborhoods, or rural districts, cities or rural regions, day care facilities, classrooms (ie, primary, elementary, or high school), colleges or universities, and workplaces. Data were analyzed between May 5, 2020, and October 20, 2020.

**Exposures:**

School reopening on September 15, 2020, vs schools remaining closed under different scenarios for nonpharmaceutical interventions.

**Main Outcomes and Measures:**

Incident and cumulative COVID-19 cases between September 1, 2020, and October 31, 2020.

**Results:**

Among 1 000 000 simulated individuals, the percentage of infections among students and teachers acquired within schools was less than 5% across modeled scenarios. Incident COVID-19 case numbers on October 31, 2020, were 4414 (95% credible interval [CrI], 3491-5382) cases in the scenario with schools remaining closed and 4740 (95% CrI, 3863-5691) cases in the scenario for schools reopening, with no other community-based nonpharmaceutical intervention. In scenarios with community-based nonpharmaceutical interventions implemented, the incident case numbers on October 31 were 714 (95% CrI, 568-908) cases for schools remaining closed and 780 (95% CrI, 580-993) cases for schools reopening. When scenarios applied the case numbers observed in early October in Ontario, the cumulative case numbers were 777 (95% CrI, 621-993) cases for schools remaining closed and 803 (95% CrI, 617-990) cases for schools reopening. In scenarios with implementation of community-based interventions vs no community-based interventions, there was a mean difference of 39 355 cumulative COVID-19 cases by October 31, 2020, while keeping schools closed vs reopening them yielded a mean difference of 2040 cases.

**Conclusions and Relevance:**

This decision analytical modeling study of a synthetic population of individuals in Ontario, Canada, found that most COVID-19 cases in schools were due to acquisition in the community rather than transmission within schools and that the changes in COVID-19 case numbers associated with school reopenings were relatively small compared with the changes associated with community-based nonpharmaceutical interventions.

## Key Points

**Question:**

What is the association of school reopening or closure with incident and cumulative COVID-19 case numbers compared with other community-based nonpharmaceutical interventions?

**Findings:**

In this decision analytical modelling study of a synthetic population of 1 000 000 individuals in Ontario, Canada, compared with community-based nonpharmaceutical interventions, school closure was associated with a small change in estimated COVID-19 incidence trajectories and cumulative case counts.

**Meaning:**

These findings suggest that community-based interventions to reduce COVID-19 case counts should take precedence over school closure.

## Teaser

This decision analytical modelling study uses a simulated population of people in Ontario, Canada, to estimate COVID-19 case counts associated with school closures and reopenings vs community-based nonpharmaceutical interventions.

## Introduction

During the first wave of the COVID-19 pandemic, school closures were a component of nonpharmaceutical interventions (NPIs) enacted to mitigate the transmission of SARS-CoV-2, largely based on the rationale that it had been effective in delaying or reducing the peak of the 2009 H1N1 influenza epidemic.^1^ It was speculated that school-aged children infected with SARS-CoV-2, who are less likely to manifest symptoms,^2^ may unknowingly pass on infections acquired in school to the members of their household and/or individuals at high risk in the community, thereby accelerating the increase in cases and subsequent strain on health care resources. In contrast, modeling studies conducted early in the course of the COVID-19 pandemic suggested that school closure was effective when combined with other NPIs, but that the effect of closure per se was modest in delaying peak case numbers or reducing the size of the peak.^3,4,5^ However, there is a high degree of uncertainty in these results, primarily owing to inability to decouple school closure from other concurrent NPIs.

School closures may affect students adversely in terms of loss of access to high-quality instruction, loss of school-based health and social services, and negative effects on physical and emotional well-being.^6,7,8,9^ Closures have been shown to result in adverse economic consequences for families, including loss of work hours to care for children and/or increased child-care expenses, which predominantly affect lower-income households.^6,7,8,9,10^

In Ontario, Canada, after closure on March 15, 2020, schools reopened on September 15, 2020. As in many jurisdictions, the second wave of COVID-19 in the province required the reinstitution of community-based NPIs and consideration of school closures. Given the negative consequences of school closure, determining the magnitude of the benefit of this measure in terms of case numbers is critical. The objective of this study is to determine the relative size of the increase in COVID-19 case numbers attributable to school reopening relative to community-based NPIs in Ontario, Canada, using an agent-based modeling approach informed by observed Ontario data.

## Methods

This study was conducted under the blanket approval of the University of Toronto Research Ethics Board for studies concerning the COVID-19 pandemic and making use of government registries. This study adheres to the Consolidated Health Economic Evaluation Reporting Standards (CHEERS) reporting guideline for modeling studies (eMethods in the Supplement).

### Development of a Synthetic Population Representative of Ontario, Canada

We developed a synthetic population of 1 000 000 individuals representative of the inhabitants of Ontario, Canada, which has a population of approximately 14.5 million people, based on data within the Social Policy Simulation Database/Model (SPSD/M) (eMethods and eTable 1 in the Supplement)^11^ developed by Statistics Canada. The SPSD/M contains data on Ontario households categorized by rural or urban location, numbers of household members, age and sex of household members, and labor force participation and industry. For the synthetic population, households were randomly sampled from the available household types to yield a total of 1 000 000 hypothetical individuals. Households were then randomly assigned to cities or a rural region according to the SPSD/M urban/rural designation of the associated household type. Within urban settings, households were further randomly assigned to neighborhoods while rural household were assigned to districts.

All members of the synthetic population spent time each day in their households, neighborhoods or districts, and their city or rural region. Additionally, children ages 2 to 3 years were assigned to daycare settings (capped at 10 children), children ages 4 to 13 years were assigned to primary or elementary schools (capped at 23 children per classroom and 150 children per school), and children ages 14 to 17 were assigned to high schools (capped at 15 children per classroom and 150 children per school). Each daycare or classroom was assigned a teacher who was randomly sampled from adults in the region whose SPSD/M industry designation was educational. Adults ages 18 to 34 years could join the workforce or potentially go to college or university (a postsecondary institution). Adults ages 18 to 34 years not enrolled in a postsecondary institution and those ages 35 to 64 years could be members of the workforce. Workplaces were characterized by industry type, region, and workplace size (ie, 1-20, 21-99, 100-499, and ≥500 workers). Working-age adults were then randomly assigned to workplaces in their region according to their SPSD/M industry designator. Adults ages 65 years and older were assumed to be retired from the workforce.

### Agent-Based Model

The agent-based model for COVID-19 transmission (ABMCT) was structured to represent the COVID-19 pandemic in Ontario and made use of the synthetic population developed for this study(eMethods and eFigure 1 in the Supplement).^11,12,13,14,15,16,17,18,19,20,21^ For the COVID-19 first wave, the model was seeded with 150 individuals who were infectious, selected from the synthetic population at random. All other individuals were initially susceptible to COVID-19 and could contact and be exposed to individuals who were infectious within their household, at school, at college or university, at workplaces, in neighborhoods and cities, or in rural districts. Exposed individuals may not have become infected and therefore remained susceptible, or they may have become infected. Individuals who were infected were unable to transmit the virus during a 4-day latent period, after which, individuals who had been infected became infectious and able to transmit the virus. Individuals who were infectious may or may not have developed symptoms. Individuals who were symptomatic entered a 1-day presymptomatic stage followed by a symptomatic stage. The duration of infectiousness for individuals with or without symptoms was modeled to be 15 days. The proportion of symptomatic cases by 10-year age group was obtained from Ontario’s Case and Contact Management Plus (CCM Plus) database (eMethods and eTable 2 in the Supplement).

Symptomatic individuals could seek health care, be confirmed as cases, and either be admitted to hospital or sent home to quarantine until recovered. Symptomatic individuals who did not seek health care could self-isolate until recovery. Self-isolated or quarantined individuals could still transmit SARS-CoV-2 to their household members. A proportion of asymptomatic cases would be detected through contact tracing, reported as confirmed cases, and quarantined until recovery. Individuals who were symptomatic but sought neither health care nor testing and undetected individuals who were asymptomatic would be free to transmit virus in household and nonhousehold settings. The probability of detection (ie, of an individual who was infectious being a confirmed case) during the first wave of COVID-19 in Ontario was estimated separately for symptomatic and asymptomatic cases via the cumulative number of confirmed cases until June 9, 2020, and the seroprevalence of antibodies for SARS-CoV-2 reported by Public Health Ontario until that date.^12^ Death outside of hospitals, such as in a long-term care facility, was not considered in the current iteration of the model, nor was nosocomial transmission within hospitals.

Susceptible individuals were modeled to be potentially infected via close contact with an individual who was infectious. Mixing of individuals was assumed to occur randomly within the included settings based on the mean number of close contacts per day prior to the institution of NPIs in March and April 2020 from a contact survey, the CONNECT study^13^ (Table 1; eMethods and eFigures 2-4 in the Supplement). We calibrated the model separately to observed COVID-19 first-wave data as a validation of the model (Table 1; eMethods, eTable 3, and eFigures 5-7 in the Supplement) and to second-wave data in September 2020 to generate subsequent confirmed case estimations according to school opening and community NPI status (Table 1; eMethods and eFigures 8-13 in the Supplement).

**Table 1.** Key Input Parameters for the Agent-Based Model for COVID-19 Transmission

Parameter	Value	Source
COVID-19 epidemiological characteristics, d		
Latent period	4	Bi et al (2020),^14^ Gostic et al (2020)^15^
Incubation period	5	Kucharski et al (2020)^16^
Infectious period	15	Voinski et al (2020)^17^
Daily contact No.		
Household, range	1-7	SPSD/M database^11^
Neighborhood/district, mean (SD)^a^	1.5 (1.1)	Brisson et al (2020)^13^
Region, mean (SD)^a^	1.8 (1.2)	Brisson et al (2020)^13^
School, mean (SD)^a^	5 (1.4)	Brisson et al (2020)^13^
Work, mean (SD)^a^	10 (1.6)	Brisson et al (2020)^13^
College or university campus	15	Calibrated value^b^
Proportionate reduction in nonhousehold contacts		
Aafter March 7, 2020, compared with prepandemic (first wave)	0.46	Calibrated value^b^
Between April 8 and July 7, 2020, compared with prepandemic (first wave)	0.36	Calibrated value^b^
Until October 1, 2020, compared with pre-pandemic (second wave)	0.81	S. Mishra, MD, PhD, instant messaging application, September 2, 2020
After October 1, 2020, compared with pre-pandemic (second wave)	0.4	Recalibrated value^b^
Household Transmission probability per contact		
Children aged ≤10 y (first wave)	0.027	Calibrated value^b^
Children aged >10 y (first wave)	0.13	Li et al (2020),^18^ Kucharski et al (2020),^16^ Bi et al (2020),^14^ Cheng et al (2020)^14^ refined via calibration^b^
Adults (first wave)	0.129	Kucharski et al (2020),^16^ Bi et al (2020),^14^ Chen et al (2020)^18^ refined via calibration^b^
Other settings (first wave)	0.037	Kucharski et al (2020),^16^ Bi et al (2020),^14^ Chen et al (2020)^18^ refined via calibration^b^
Children aged ≤10 y (second wave)	0.024	Recalibrated value^b^
Children >10 y (second wave)	0.116	Li et al (2020),^18^ Kucharski et al (2020),^16^ Bi et al (2020),^14^ Cheng et al (2020)^19^ refined via recalibration^b^
Adults (second wave)	0.115	Kucharski et al,^16^ Bi et al,^14^ Cheng et al^19^ Refined via recalibration^b^
Other settings (second wave)	0.033	Kucharski et al (2020),^16^ Bi et al (2020),^14^ Cheng et al (2020)^19^ refined via re-calibration^b^
Proportionate reduction in transmissibility		
Owing to asymptomatic status	0.90	Kucharski et al (2020)^16^ refined via calibration^b^
After March 7, 2020 (first wave)	0.46	Calibrated value^b^
After April 8, 2020 (first wave)	0.36	Calibrated value^b^
After Oct 1., 2020 (second wave)^c^	0.5	Recalibrated value^b^
Case detection probability		
Symptomatic (first wave)	0.196	Tuite et al (2020),^20^ PHO^12^ refined via calibration^b^
Asymptomatic (first wave)	0.140	Tuite et al (2020),^20^ PHO^12^ refined via calibration^b^
Symptomatic	0.287	Tuite et al (2020),^20^ PHO^12^ refined via re-calibration^b^
Asymptomatic	0.187	Tuite et al (2020),^20^ PHO^12^ refined via recalibration^b^
Mitigating factors		
Probability of self-isolation among symptomatic cases	0.90	Kucharski et al (2020)^16^
Masks vs no masks, odds ratio (95% CI)	0.15 (0.07-0.34)	Chu et al (2020)^21^

Abbreviation: PHO, Public Health Ontario.

^a^ SD of the log contact number assumed to be 0.2 × mean log contact number with an assumed correlation coefficient among settings of 0.9.

^b^ Calibration refers to adjustment of the model input values derived from jurisdictions other than Ontario or determining the most likely input value for parameters unavailable from data or literature sources to minimize the difference between modeled and observed daily new confirmed COVID-19 cases in the first wave of the pandemic. Recalibration refers to a similar process for adjusting the input parameters of the agent-based model for September 2020. The recalibrated model was then used to generate estimates for school opening and community-based nonpharmaceutical interventions in September and October 2020.

^c^ In scenarios in which nonpharmaceutical interventions were implemented on October 1, 2020.

### Daycare and School Opening Scenarios

We simulated 2 opening scenarios, (eTable 4 in the Supplement). First, we modeled a counterfactual scenario in which daycares and schools did not reopen on September 15, 2020 (scenario A). Second, we modeled a scenario in which schools and daycares reopened on September 15, 2020, (scenario B) but with several measures in place to limit within-school transmission of COVID-19: daycare centers were capped at 10 children, primary and elementary class sizes were capped at 23 students, and high school classes were capped at 15 students; students remained in their assigned classrooms for the school day rather than moving among classrooms; universal masking was in place; in designated high schools in urban areas, students attended school only on alternate weekdays; and if more than 2 confirmed cases of COVID-19 occurred in a daycare or classroom less than 2 weeks apart, the daycare or classroom was closed for 14 days, with the children in the class excluded from school rather than moved to another classroom (eTable 4 in the Supplement).

### NPI Scenarios

We modeled 3 community-based NPI scenarios (eTable 4 in the Supplement) in Ontario at the beginning of October 2020 in response to increasing confirmed daily case incidence from 185 cases on September 1 to 675 cases on September 30 (eFigures 8-10 in the Supplement). First, we modeled a scenario in which no additional NPIs were enacted so that the increase in case numbers observed in September continued, unabated, until October 31, 2020 (scenario 1). Second, we modeled a scenario in which community-based NPIs were enacted on October 1, 2020, that resulted in a reduction in contacts outside of households to 40% of the value observed prepandemic, including closing of workplaces to nonessential workers, and a reduction in the probability of transmission per contact of 50% compared with the recalibrated values within household and other settings (scenario 2). Third, we modeled a scenario in which the increase of new infections was limited to 0.8% per day, replicating the reduced increase of confirmed cases in the CCM plus database for the first 15 days of October (scenario 3) (eTable 4 in the Supplement).

### Statistical Analysis

The 2 school reopening and 3 community-based NPI scenarios created 6 combinations, 1A to 3B (eTable 4 in the Supplement). For each combination, we ran 100 repetitions of the ABMCT. In addition, 2 deterministic 1-way sensitivity analyses were conducted using the 2A and 2B parameter sets and either allowing the effectiveness of within-school mitigation to vary or the relative effectiveness of community NPIs to vary. All analyses were conducted with TreeAge Pro statistical software version 2020 R2 (TreeAge). Data were analyzed from May 5 to October 20, 2020.

## Results

All results are presented on a provincial scale: case numbers derived from the 1 000 000-person synthetic population were multiplied by 14.5). The simulated population had a mean (SD) age of 41.5 (23.4) years, and 507 304 (50.7%) were women.

### Classroom Closures

The median (interquartile range [IQR]) numbers of classroom closures for scenarios involving school reopening was estimated to be 1726 (1523-1943) classrooms with lack of community-based NPI implementation (scenario 1B), 1051 (957-1149) classrooms with NPI implementation (scenario 2B), and 587 (493-671) classrooms with replication of observed COVID-19 case counts in early October (scenario 3B) (Table 2; eFigure 14 and eFigure 15 in the Supplement), with most closures occurring in primary and elementary schools. For teachers and students, median (IQR) numbers of infections acquired within schools were estimated to be 493 (435-569) infections in scenario 1B, 421 (373-482) infections in scenario 2B, and 167 (145-203) infections in scenario 3B, while the median (IQR) numbers acquired in the community were 15 892 (14 837-16 679) infections in scenario 1B, 9643 (9008-10 415) infections in scenario 2B, and 7265 (6982-7830) infections in scenario 3B, corresponding to median (IQR) percentages of total infections acquired in schools estimated at 3.15% (2.79%-3.55%) in scenario 1B, 4.19% (3.80%-4.60%) in scenario 2B, and 2.37% (2.02%-2.86%) in scenario 3B (Table 2; eFigure 14 and eFigure 15 in the Supplement) among an estimated 307 921 daycare children and staff, 1 562 402 elementary school students and staff, and 713 948 high school students and staff.^22,23^

**Table 2.** Model Simulation Output for Estimated Number of Classroom Closures and Location of Acquisition of Infection

Simulation output value	Estimated cases, median (IQR), No.^a^		
	No NPIs imposed (scenario 1B)	NPIs imposed (scenario 2B)	Replicating observed counts (scenario 3B)
Any classroom/daycare closures	1726 (1523-1943)	1051 (957-1149)	587 (493-671)
Daycare closures	87 (58-102)	44 (29-58)	15 (12-29)
Primary or elementary classroom closures	1175 (1044-1323)	725 (653-812)	406 (348-464)
High school classroom closures	479 (388-566)	276 (232-319)	160 (131-203)
SARS-CoV-2 infections acquired in school	493 (435-569)	421 (373-482)	167 (145-203)
SARS-CoV-2 infections acquired outside of school	15 892 (14 837-16 679)	9643 (9008-10 415)	7265 (6982-7830)
Estimated SARS-CoV-2 infections acquired in school, %	3.15 (2.79-3.55)	4.19 (3.80-4.60)	2.37 (2.02-2.86)

Abbreviations: IQR, interquartile range; NPI, non-pharmaceutical intervention.

^a^ Results of simulations are shown for scenarios (1B, 2B, and 3B; eTable 4 in the Supplement) in which schools were modeled to reopen on September 15, 2020. Estimated median values and IQRs were obtained from 100 model repetitions for each scenario. Estimated numbers of confirmed infections correspond to those among children in daycare (ages 2-3 years), primary and elementary school (ages 4-13 years), or high school (ages 14-17 years) from September 1 to October 31, 2020. Estimated infections and classroom closure numbers are on the Ontario provincial scale of 14.5 million individuals.

### Daily New Confirmed COVID-19 Cases

**Figure 1.**
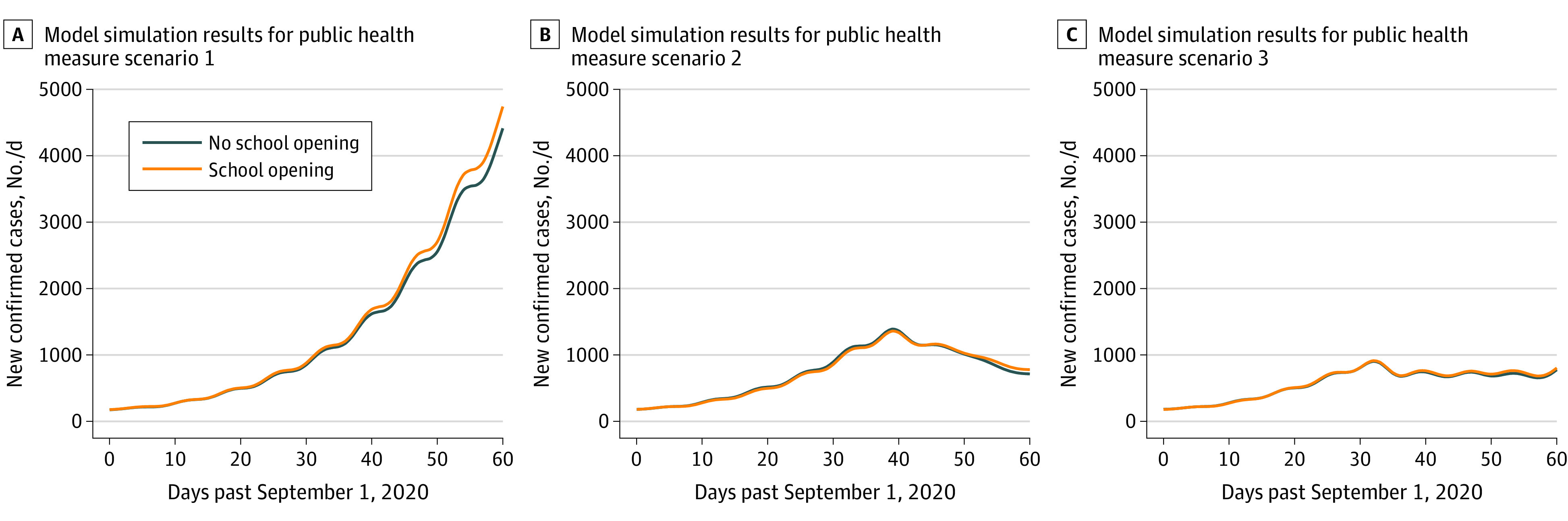
Mean of 100 Model Repetitions for Scenarios in Which Schools Reopened or Remained Closed on September 15, 2020 Modeled counts subjected to Gaussian kernel smoothing with a bandwidth of 2 days. Scenario 1 indicates no public health interventions restricting contacts or reducing the probability of transmission of COVID-19 between contacts implemented (ie, if the trends in the rise of new cases in September had been allowed to persist through October); scenario 2, implementation of public health interventions restricting contacts and reducing the probability of transmission of COVID-19 between contacts implemented (ie, if the trends in the increase of new cases in September had not been allowed to persist through October); scenario 3, the slowing of the rate of growth of cases observed from October 1 to 15, 2020, to 0.8% per day, persisted until October 31, 2020.

When community-based NPIs were not implemented (ie, scenarios 1A and 1B), the mean number of daily new confirmed cases on October 31, 2020, were estimated to be 4414 (95% credible interval [CrI], 3491-5382) cases with schools closed (scenario 1A) vs 4740 (95% CrI, 3863-5691) cases with schools reopened (scenario 1B) (Figure 1A; eTable 5, eFigure 16, and eFigure 17 in the Supplement). If community-based NPIs were implemented (scenarios 2A and 2B), the mean number of daily new confirmed cases on October 31, 2020, was estimated to be 714 (95% CrI, 568-908) cases with schools closed (scenario 2A) vs 780 (95% CrI, 580-993) cases with schools reopened (scenario 2B) (Figure 1B; eTable 5, eFigure 18, and eFigure 19 in the Supplement). For the scenarios in which the change in the increase of observed case counts in early October 2020 was replicated (scenarios 3A and 3B), the mean number of daily new confirmed cases on October 31, 2020, was estimated to be 777 (95% CrI, 621-993) cases with schools closed (scenario 3A), vs 803 (95% CrI, 617-990) cases with schools reopened (scenario 3B) (Figure 1C; eTable 5, eFigure 20, and eFigure 21 in the Supplement).

### Cumulative Confirmed COVID-19 Cases

**Figure 2.**
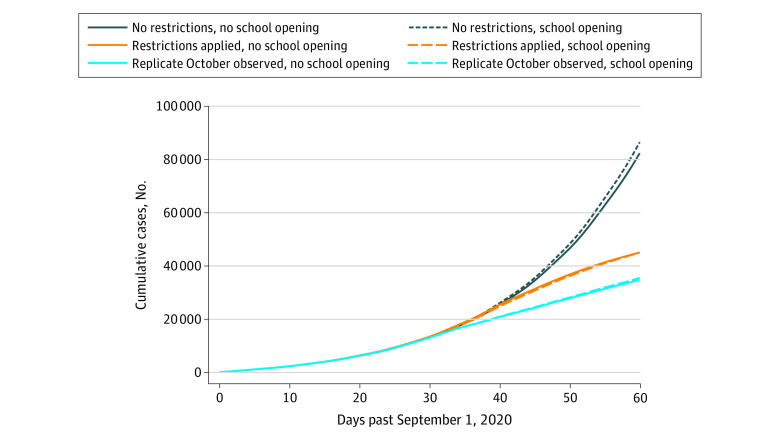
Comparison of Model Simulations for the Estimated Cumulative Number of COVID-19 Cases in Ontario Between September 1 and October 31, 2020, Among 6 Modeled Scenarios Modeled counts subjected to Gaussian kernel smoothing with a bandwidth of 2 days.

When community-based NPIs were not implemented (scenarios 1A and 1B) the mean, cumulative number of confirmed cases by October 31, 2020, was estimated to be 82 372 (95% CrI, 64 448-102 518) cases with schools closed (scenario 1A) vs 86 507 (95% CrI, 68 624-105 505) cases with schools reopened (scenario 1B) (Figure 2; eTable 5 in the Supplement). If community-based NPIs were implemented (scenarios 2A and 2B) the mean cumulative number of cases by October 31, 2020, was estimated to be 45 112 (95% CrI, 35 873-56 790) cases with schools closed (scenario 2A) and 45 048 (95% CrI, 34 781-55 203) cases with schools reopened (scenario 2B) (Figure 2; eTable 5 in the Supplement). For the scenarios in which the change in the increase of observed case counts in early October 2020 were replicated (scenarios 3A and 3B), the mean cumulative number of cases by October 31, 2020, was estimated to be 34 911 (95% CrI, 27 302-44 127) cases when schools remained closed (scenario 3A) vs 35 581 (95% CrI, 28 278-43 986) cases with schools reopened (scenario 3B) (Figure 2; eTable 5 in the Supplement). The mean difference in cumulative COVID-19 cases by October 31, 2020, for scenarios in which community-based NPIs were not implemented (scenarios 1A and 1B) vs scenarios in which NPIs were implemented (scenarios 2A and 2B) was estimated to be 39 355 cases (eFigure 22 in the Supplement). In contrast, the mean difference in cumulative COVID-19 cases by October 31, 2020, for the scenarios in which schools were reopened (scenarios 1B and 2B) vs scenarios in which they were not (scenarios 1A and 2A) was estimated to be 2040 cases (eFigure 22 in the Supplement).

### Sensitivity Analyses

When NPIs were implemented and their effectiveness held at the base case value, as the effectiveness of mitigation efforts within schools diminished, the difference in mean estimated cumulative case numbers by October 31, 2020, between keeping schools closed or reopening them increased (eFigure 23 in the Supplement). When school mitigation effectiveness was held at the base case value, as the effectiveness of community-based NPIs decreased, the difference in mean estimated cumulative case numbers between keeping schools closed vs reopening them did not increase (eFigure 24 in the Supplement).

## Discussion

The findings of our decision analytical modelling study indicate that school reopening, compared with the counterfactual situation in which they remained closed on September 15, 2020, was associated with an increase in incident and cumulative COVID-19 cases across 3 community-based NPI implementation scenarios in Ontario, Canada, between September 1 and October 31, 2020, but most infections among students and staff were estimated to be acquired in the community rather than within schools. In our simulations, we show that community-based NPIs directed at reducing contacts, such as restricting gatherings, limiting workplaces to essential workers, and reducing transmission between contacts (eg, by requiring mask-wearing), had a much larger effect in our simulations on reducing incident or cumulative COVID-19 cases than keeping schools closed vs reopening them. It is important to note that our model assumed that measures would be taken in schools to mitigate spread of the virus by reducing high school class sizes, having students remain in the same classroom rather than moving among them, requiring universal mask-wearing, and closing classrooms when more than 2 confirmed SARS-CoV2 infections occurred within any 2-week period.

The results of our simulations are in agreement with a meta-analysis of COVID-19 pandemic data in Hong Kong, Singapore, and mainland China conducted by Viner at al,^24^ who found that school closures did not materially contribute to the prevention of population spread. Likewise, in Denmark, the first country in Europe to reopen schools, the incidence of COVID-19 continued to decline after reopening of day care facilities and elementary schools in mid-April and middle and high schools in May 2020, albeit with stringent physical distancing measures in place.^25^ Likewise, cross-sectional studies in North Carolina^26^ and the United Kingdom^27^ found low numbers of within-school transmissions (32 in North Carolina and 133 in the United Kingdom) among sizable cohorts (90 000 students attending per day in North Carolina and 928 000 students attending per day in the United Kingdom). A study in Wisconsin by Falk et al^28^ found that, among COVID-19 cases identified in schools, 3.7% were due to within-school transmission, which is similar in magnitude to our estimated percentages from the ABMCT. However, it is worth noting that observational studies reporting symptomatic cases may underestimate the true rate of within-school transmission by missing students who are asymptomatic.^29^

Our results were also similar to several prior modeling studies.^3,4,5^ However, these studies^3,4,5^ were conducted early in the course of the pandemic when precise information regarding transmission probability between cases and when observational data required for model calibration were unavailable or did not distinguish between infections acquired in school vs those acquired in the community and imported into schools. Our results bolster the prior studies^3,4,5^ but considered events later in the course of the pandemic, at the traditional time of school reopening in Canada, and made use of accumulated observational calibration data.

Our simulations showing that most infections in schools were associated with community acquisition rather than transmission within schools are congruent with numerous contact tracing studies of secondary infections in schools conducted in many jurisdictions, which show very infrequent onward transmission within schools.^30,31,32,33,34,35,36^ For example, in a systematic review, Public Health Ontario^37^ synthesized 4 studies of outbreak investigations that showed that 28 index cases among students and teachers lead to 2092 close contacts from which there were only 2 transmissions. Forbes et al,^38^ in an analysis of health services data in the United Kingdom involving more than 9 million adults younger than 65 years, reported that living with a child aged 11 years or younger was not associated with increased SARS-CoV-2 infections or with COVID-19 ward or intensive care unit admissions. Furthermore, data obtained in the United Kingdom showed a strong correlation between the frequency of school outbreaks and regional COVID-19 incidence.^39^

Our simulation results suggest that the relatively modest estimated reduction in COVID-19 cases derived from keeping schools closed should be balanced against the adverse effects on children and families. School closure may interfere with educational advancement, prevent access to school-based health and social programs, reduce physical activity among children, and negatively affect household finances, particularly among low income households.^10^

### Limitations and Strengths

Our study has limitations. Like all models, our ABMCT is a simplification of complex, dynamic patterns of interactions and viral transmission in real populations. For example, although close contacts may occur among students, particularly for adolescents, just before and after school hours, this was not explicitly modeled. However, this mixing was partially captured in the close contacts among individuals in neighborhoods or rural districts. Modeled NPI scenarios may not precisely reflect the actual implementation of public health policy in Ontario but have the advantage of quantitatively determining the outcomes associated with school reopenings across a range of community-based NPI possibilities with robust qualitative results. Also, by not being overly jurisdiction-specific, the results of our ABMCT may be broadly applicable to regions similar to Ontario in terms of school systems, populations, and economy. Latent, incubation, and infectious periods were assumed to be fixed rather than drawn from distributions, which may have caused an underestimation of the uncertainty of pandemic trajectories but would not have affected the mean results. The ABMCT did not explicitly consider COVID-19 testing volumes as a potential driver of confirmed case numbers but rather assumed that, over time, testing rates, and therefore case detection probability, would stabilize.

Our study also has several strengths. The use of a synthetic population allows for realistic linkages of individuals into discrete clusters, such as households, classrooms, and workplaces, unlike other approaches that treat mixing of individuals among age strata in a probabilistic fashion. The agent-based modeling approach allows for so-called *super-spreading events*, in which a small number of individuals have a disproportionately large number of close contacts, and for representation of relatively complicated measures, such as classroom closure after a certain number of confirmed cases over a particular interval, which would be difficult with other transmission model designs, such as compartmental models. Unlike previous transmission models, we had the advantage of being able to calibrate our model to observational data on case numbers in Ontario from March through September 2020. Agent-based modeling allows the source of infections to be determined allowing the disentanglement of infections transmitted in schools vs those acquired in the community and brought into the school setting.

## Conclusions

The results of this decision analytical modeling estimated that the magnitude of the effect of schools being open on COVID-19 cases was substantially lower than the effect of community-based NPIs. These findings suggest that school closure be considered the last resort in the face of a resurgence of COVID-19, and that efforts should instead focus on widespread reduction of community transmission.

## Notes

Supplement. **eMethods.** Supplemental Methods **eTable 1.** Comparison of Characteristics of the Synthetic Population Employed in Simulations and the Corresponding Values for the Ontario Population **eTable 2.** Logistic Coefficients for Imputation of Missing Symptomatic Status and for Assignment of Symptomatic Status of Individuals Infected With SARS-CoV-2 in the Agent Based Model of COVID-19 Transmission (ABMCT) **eTable 3.** Percentage of Essential Workers by Social Policy Simulation Database and Model (SPSD/M) Industry Designation **eTable 4.** Summary of Simulation Scenarios **eTable 5.** Summary of Main Simulation Results **eFigure 1.** Agent-Based Model of COVID-19 Transmission (ABMCT) Model Schematic **eFigure 2.** Demonstration of 5000 Samples From the Multivariate Lognormal Distribution of Daily Contacts at Work **eFigure 3.** Demonstration of 5000 Samples From the Multivariate Lognormal Distribution of Potential Numbers of Daily Contacts at Day Care and Primary, Elementary, and High Schools **eFigure 4.** Demonstration of the Correlation of 5000 Samples From the Multivariate Lognormal Distribution of Potential Numbers of Daily Contacts at Work and Within the Urban City or Rural Region **eFigure 5.** Observed vs Smoothed Daily New Symptomatic COVID-19 Case Counts (First Wave) for Various Gaussian Kernel Smoothing Bandwidths **eFigure 6.** Observed vs Smoothed Daily New Symptomatic COVID-19 Case Counts (First Wave) for the Selected Gaussian Kernel Smoothing Bandwidth of 7 Days **eFigure 7.** Model Calibration to Daily New Symptomatic Confirmed COVID-19 Cases in Ontario (First Wave) **eFigure 8.** Observed vs Smoothed Daily New Symptomatic COVID-19 Case Counts (Second Wave) for Various Gaussian Kernel Smoothing Bandwidths **eFigure 9.** Observed vs Smoothed Daily New Symptomatic COVID-19 Case Counts (Second Wave) for the Selected Gaussian Kernel Smoothing Bandwidth of 2 Days **eFigure 10.** Model Calibration to Daily New Total Confirmed COVID-19 Cases in Ontario (Second Wave) **eFigure 11.** Observed vs Smoothed Daily New Symptomatic COVID-19 Case Counts During the First 15 Days of October 2020, for Various Gaussian Kernel Smoothing Bandwidths **eFigure 12.** Observed vs Smoothed Daily New Symptomatic COVID-19 Case Counts During the First 15 Days of October 2020, for the Selected Gaussian Kernel Smoothing Bandwidth of 2 Days **eFigure 13.** Model Calibration to Daily New Total Confirmed COVID-19 Cases in Ontario During the First 15 Days of October 2020 **eFigure 14.** Distribution of Numbers of Day Care, Primary, Elementary and High School Classroom Closures for Scenarios in Which Schools Had Reopened **eFigure 15.** Distribution of the Percentage of SARS-CoV-2 Infections Acquired in Schools for Students and Teachers in Scenarios in Which Schools Had Reopened **eFigure 16.** Model Simulation Results for Scenario 1A **eFigure 17.** Model Simulation Results for Scenario 1B **eFigure 18.** Model Simulation Results for Scenario 2A **eFigure 19.** Model Simulation Results for Scenario 2B **eFigure 20.** Model Simulation Results for Scenario 3A **eFigure 21.** Model Simulation Results for Scenario 3B **eFigure 22.** Reduction of Cumulative Cases Between September 1 and October 31, 2020, Attributable to the 2 Policy Choices to Implement Public Health Restrictions or Not vs to Open Schools or Not **eFigure 23.** Sensitivity Analysis for Effectiveness of School-Based Mitigation of SARS-CoV-2 Transmission **eFigure 24.** Sensitivity Analysis for Effectiveness of Community-Based, Nonpharmaceutical Interventions **eReferences**
