# Supplementary material for: Simulation-Based Estimation of SARS-CoV-2 Infections Associated With School Closures and Community-Based Nonpharmaceutical Interventions in Ontario, Canada
Source: JAMA Netw Open. 2021 Mar 31;4(3):e213793. doi: 10.1001/jamanetworkopen.2021.3793 (PMC8013816; doi:10.1001/jamanetworkopen.2021.3793)
Supplement: Supplement. — eMethods. Supplemental Methods eTable 1. Comparison of Characteristics of the Synthetic Population Employed in Simulations and the Corresponding Values for the Ontario Population eTable 2. Logistic Coefficients for Imputation of Missing Symptomatic Status and for Assignment of Symptomatic Status of Individuals Infected With SARS-CoV-2 in the Agent Based Model of COVID-19 Transmission (ABMCT) eTable 3. Percentage of Essential Workers by Social Policy Simulation Database and Model (SPSD/M) Industry Designation eTable 4. Summary of Simulation Scenarios eTable 5. Summary of Main Simulation Results eFigure 1. Agent-Based Model of COVID-19 Transmission (ABMCT) Model Schematic eFigure 2. Demonstration of 5000 Samples From the Multivariate Lognormal Distribution of Daily Contacts at Work eFigure 3. Demonstration of 5000 Samples From the Multivariate Lognormal Distribution of Potential Numbers of Daily Contacts at Day Care and Primary, Elementary, and High Schools eFigure 4. Demonstration of the Correlation of 5000 Samples From the Multivariate Lognormal Distribution of Potential Numbers of Daily Contacts at Work and Within the Urban City or Rural Region eFigure 5. Observed vs Smoothed Daily New Symptomatic COVID-19 Case Counts (First Wave) for Various Gaussian Kernel Smoothing Bandwidths eFigure 6. Observed vs Smoothed Daily New Symptomatic COVID-19 Case Counts (First Wave) for the Selected Gaussian Kernel Smoothing Bandwidth of 7 Days eFigure 7. Model Calibration to Daily New Symptomatic Confirmed COVID-19 Cases in Ontario (First Wave) eFigure 8. Observed vs Smoothed Daily New Symptomatic COVID-19 Case Counts (Second Wave) for Various Gaussian Kernel Smoothing Bandwidths eFigure 9. Observed vs Smoothed Daily New Symptomatic COVID-19 Case Counts (Second Wave) for the Selected Gaussian Kernel Smoothing Bandwidth of 2 Days eFigure 10. Model Calibration to Daily New Total Confirmed COVID-19 Cases in Ontario (Second Wave) eFigure 11. Observed vs Smoothed Daily New Symptomatic C [file jamanetwopen-e213793-s001.pdf]

# Supplemental Online Content

Naimark D, Mishra S, Barrett K, et al. Simulation-Based Estimation of SARS-CoV-2 Infections Associated With School Closures and Community-Based Nonpharmaceutical Interventions in Ontario, Canada. *JAMA Netw Open*. 2021;4(3):e213793. doi:10.1001/jamanetworkopen.2021.3793

**eMethods.** Supplemental Methods
**eTable 1.** Comparison of Characteristics of the Synthetic Population Employed in Simulations and the Corresponding Values for the Ontario Population
**eTable 2.** Logistic Coefficients for Imputation of Missing Symptomatic Status and for Assignment of Symptomatic Status of Individuals Infected With SARS-CoV-2 in the Agent Based Model of COVID-19 Transmission (ABMCT)
**eTable 3.** Percentage of Essential Workers by Social Policy Simulation Database and Model (SPSD/M) Industry Designation
**eTable 4.** Summary of Simulation Scenarios
**eTable 5.** Summary of Main Simulation Results
**eFigure 1.** Agent-Based Model of COVID-19 Transmission (ABMCT) Model Schematic
**eFigure 2.** Demonstration of 5000 Samples From the Multivariate Lognormal Distribution of Daily Contacts at Work
**eFigure 3.** Demonstration of 5000 Samples From the Multivariate Lognormal Distribution of Potential Numbers of Daily Contacts at Day Care and Primary, Elementary, and High Schools
**eFigure 4.** Demonstration of the Correlation of 5000 Samples From the Multivariate Lognormal Distribution of Potential Numbers of Daily Contacts at Work and Within the Urban City or Rural Region
**eFigure 5.** Observed vs Smoothed Daily New Symptomatic COVID-19 Case Counts (First Wave) for Various Gaussian Kernel Smoothing Bandwidths
**eFigure 6.** Observed vs Smoothed Daily New Symptomatic COVID-19 Case Counts (First Wave) for the Selected Gaussian Kernel Smoothing Bandwidth of 7 Days
**eFigure 7.** Model Calibration to Daily New Symptomatic Confirmed COVID-19 Cases in Ontario (First Wave)
**eFigure 8.** Observed vs Smoothed Daily New Symptomatic COVID-19 Case Counts (Second Wave) for Various Gaussian Kernel Smoothing Bandwidths
**eFigure 9.** Observed vs Smoothed Daily New Symptomatic COVID-19 Case Counts (Second Wave) for the Selected Gaussian Kernel Smoothing Bandwidth of 2 Days
**eFigure 10.** Model Calibration to Daily New Total Confirmed COVID-19 Cases in Ontario (Second Wave)
**eFigure 11.** Observed vs Smoothed Daily New Symptomatic COVID-19 Case Counts During the First 15 Days of October 2020 for Various Gaussian Kernel Smoothing Bandwidths

© 2021 Naimark D et al. *JAMA Network Open.*

**eFigure 12.** Observed vs Smoothed Daily New Symptomatic COVID-19 Case Counts During the First 15 Days of October 2020 for the Selected Gaussian Kernel Smoothing Bandwidth of 2 Days
**eFigure 13.** Model Calibration to Daily New Total Confirmed COVID-19 Cases in Ontario During the First 15 Days of October 2020
**eFigure 14.** Distribution of Numbers of Day Care, Primary, Elementary and High School Classroom Closures for Scenarios in Which Schools Had Reopened
**eFigure 15.** Distribution of the Percentage of SARS-CoV-2 Infections Acquired in Schools for Students and Teachers in Scenarios in Which Schools Had Reopened
**eFigure 16.** Model Simulation Results for Scenario 1A
**eFigure 17.** Model Simulation Results for Scenario 1B
**eFigure 18.** Model Simulation Results for Scenario 2A
**eFigure 19.** Model Simulation Results for Scenario 2B
**eFigure 20.** Model Simulation Results for Scenario 3A
**eFigure 21.** Model Simulation Results for Scenario 3B
**eFigure 22.** Reduction of Cumulative Cases Between September 1 and October 31, 2020, Attributable to the 2 Policy Choices to Implement Public Health Restrictions or Not vs to Open Schools or Not
**eFigure 23.** Sensitivity Analysis for Effectiveness of School-Based Mitigation of SARS-CoV-2 Transmission
**eFigure 24.** Sensitivity Analysis for Effectiveness of Community-Based, Nonpharmaceutical Interventions
**eReferences**

This supplemental material has been provided by the authors to give readers additional information about their work.

© 2021 Naimark D et al. *JAMA Network Open.*

**eMethods.** Supplemental Methods

## Development of a synthetic population representative of Ontario, Canada

We developed a synthetic population of one million individuals representative of the inhabitants of Ontario, Canada (population ~ 14.5 million) based on data within the Social Policy Simulation Database and Model (SPSD/M) [1] developed by Statistics Canada (eTable1).

**Selection of households**

SPSD/M contains data on households in Ontario categorized by rural or urban location, numbers of household members, the age and sex of household members, labour force participation and industry. Household types consist of the 39,774 combinations of the latter factors. Each type has an associated household weight representing the number of households in Ontario with that particular configuration. For the synthetic population, 413,957 households were randomly sampled from the available household types, according to the household weights, to yield 1 million hypothetical individuals.

**Allocation of households to cities, a rural region, urban neighborhoods and rural districts**

The geography of the model consisted of 52 named Ontario cities and one rural region (eTable 1). The populations of the cities in the 2011 and 2016 Canadian censuses were used to calculate an annual exponential rate of expansion (or contraction) which was then used to estimate the population in 2020. The latter estimates were then reduced to model scale by dividing by 14.5. The population of the rural region was estimated as the 2020 population of Ontario minus the sum of the estimated populations of the cities. The Ontario rural population was then divided by 14.5 to estimate its size on model scale.

Households were assigned to one of the cities or the rural region according to the rural/urban SPSD/M indicator associated with the household. Cities were arranged alphabetically and assignments to cities consisted of iterating through each household and adding it to the first city until the city's model-scale population was 'full' and then continuing with household iteration to fill the next city and so forth. Although households were assigned sequentially according to their household identity number, since the selection of households for each identifier was random, the allocation to cities was also random in effect. Urban households were not split between cities: if the last household to be assigned to a city would cause the population of the city to exceed the desired value by a few individuals, this was allowed.

Within urban settings, households were further randomly assigned to neighborhoods which were configured as square tiles with areas of 4 $km^2$ such that the number of individuals in a neighborhood was determined by the population density of the city. A similar process of adding households to neighborhoods sequentially as described for cities was employed to populate neighborhoods. Likewise, a similar process assigned rural household to districts that had an area of 64 $km^2$ and a population density equal to the average for rural Canada of 150/$km^2$ [2]. As was the case for cities, households were not split between neighborhoods or districts.

**Allocation of children to daycare facilities or schools**

Children less than 2 years old were assumed to remain within their household or accompany their parents into neighborhood/districts and their assigned city or rural region. Older children spent time within the household, neighborhood/district and city/region but also could potentially go to school on weekdays during the school year (subject to general school opening or closure and specific restrictions, see the main text for details). Children between 2 and 3 years old were assigned to daycare settings with a cap of 10 children per daycare, children between 4 and 13

© 2021 Naimark D et al. *JAMA Network Open.*

were assigned to primary/elementary schools with a classroom cap of 23 students and children between 14 and 17 were assigned to high schools (secondary schools) with a classroom cap of 15. Daycare centres were assumed to draw children from a single neighborhood or district. On model scale, primary/elementary and high school enrollments were limited to 150 students. These schools drew students from multiple contiguous neighborhoods or districts. Each daycare or classroom was assigned a teacher who was randomly sampled from adults in the region whose SPSD/M industry designation was 11, educational services.

**Allocation of young adults to post-secondary educational institutions**

Young adults, 18 to 34 years old, could join the workforce or potentially go to college or university (a post-secondary institution). The probability of the latter was based on the total enrollment among colleges and universities in Ontario divided by the size of the 18 to 34 year-old population in the province. The attendance of a student at a given institution was selected randomly according to the proportion of post-secondary students in Ontario enrolled at that college or university. Colleges and universities were assumed to draw students from all cities and the rural region (e.g. a student whose household was located in the city of Barrie, Ontario, could attend the York University located in Toronto). When post-secondary institutions were in session, students could spend time on campus or in the region in which the institution was located. During the summer break or during general college or university closures (which was assumed to be the case after March 15, 2020), students returned to their households and spent time in the associated neighborhood/district and city/region.

**Allocation of working-age adults to workplaces**

Adults, 18 to 34 yeas old, not enrolled in a post-secondary institution, and those 35 to 64 years old, could be members of the workforce. Workplaces were characterized by industry type, region, and workplace size. Workplace sizes were categorized as extremely small (1 – 10 workers), small (11 – 99 workers), medium (100 – 499 workers), and large (> 500 workers). Initially, since the SPSD/M has an industry identifier assigned to every individual, the number of potential workplace assignments in each combination of these three factors was obtained by taking the product of the three marginal proportions (the proportion of individuals in each region, industry and workplace size) and the synthetic population size of 1 million individuals. Then, because some combinations of industry and region yielded too few potential workplace assignments for medium and large workplaces, they were re-assigned to larger population centres. Working age adults were then randomly assigned to workplaces in their region according to their SPSD/M industry designator using the sequential fill method described for geographic units above. If a workplace could not be found for an individual within a city or region, an alternate workplace in an adjacent city was sought. If no workplaces could be identified in the back-up city, a placement was sought in the largest population centre, Toronto. If no workplace could be identified, the person was designated as unemployed and could spend time within their household, neighborhood or district, and region. Employed, working-age adults spent time at home, within their neighborhood or district and, on weekdays, at work.

**Older adults retired from the workforce**

Adults, 65 and older, were assumed to be retired from the workforce and could spend time in their households, neighborhoods or districts, and cities or rural region.

**The synthetic population as tabular data**

The synthetic population existed as a set of look-up tables in the form of a relational database. For example, individual-level attributes were stored in a table indexed by a personal identifier with one million rows and 11 columns representing attributes. One of the latter was a household

© 2021 Naimark D et al. *JAMA Network Open.*

indicator. A second table of household data was indexed by household indicator and provided the individual identifiers of the of the members of the household. Similar lookup tables were constructed for neighborhoods/districts, cities/regions, daycare facilities, schools, classrooms, universities and workplaces. In the description above, 'spending time in a particular setting', for example, a particular student spending time in a classroom within a school, should be understood to mean that the synthetic student's personal identifier was included among the set of personal identifiers of the students attending that classroom within the row corresponding to its classroom identifier in the classroom lookup table.

## ABMCT model structure and function

The Agent-based Model for COVID-19 Transmission (ABMCT) was structured to model the COVID-19 pandemic in Ontario and made use of the synthetic population described above. It followed an SEIR structure with four groups of hypothetical individuals: Susceptible persons who had not yet acquired COVID-19, infEcted persons who had acquired SARS-CoV2 infection but were not yet able to transmit the infection, Infectious persons who were able to transmit the infection and Removed persons who had either died or recovered from COVID-19.

The model was initiated with a seed-group of infectious individuals, on day 0, who were treated as active agents, and who started to potentially infect other susceptible individuals. To spare computational resources, the remainder of the individuals in either the susceptible or exposed group existed in the synthetic population's tabular data but did not exist as active agents in the ABMCT model. In other words, the ABMCT was an (S)EIR model where only persons in the infected, infectious and removed categories were actively modelled. Susceptible persons existed, essentially, as passive targets of COVID-19 infection. Whether their entries would be accessible to active infectious agents is described in the model sub-process section below. Once infected, latent individuals entered the model, were imbued with their attributes (i.e. the data stored in their tabular entries were converted to active tracking variables), and they became active agents.

The ABMCT was constructed as a parallel, open, microsimulation model with time steps equal to one day. The model was parallel in the sense that infected individuals were in the model all together and open in the sense that new infected individuals entered the model each day. Subsequent to day 0, synthetic individuals who were infected the day before entered into the microsimulation model as new active agents in the infected state (eFigure 1). After a four-day latent period (table 1 in the main text), individuals moved from the infected to the infectious state and begin contacting susceptible individuals and potentially transmitting COVID-19. During their 15-day infectious period (table 1 in the main text), agents in the model could be admitted to hospital which was considered as a transition into the removed state. At the end of 15 days, infectious individuals recovered and transitioned into the removed state.

The ABMCT consisted of the following subprocesses:

1. A model initiation sub-process–working matrices were established and filled them with initial values.
2. A prior infection sub-process – for second wave simulations, a proportion of the synthetic population was randomly selected from within 10-year age categories to have been infected prior to day 0 and no longer susceptible to COVID-19.
3. A seed infection sub-process – the number of seed individuals was randomly sampled from the synthetic population. In second wave simulations, persons removed prior to day 0 could not be selected as seed individuals.

© 2021 Naimark D et al. *JAMA Network Open.*

4. A model time to calendar time conversion sub-process – the day 0 date was set so that subsequent model day numbers could be converted into calendar dates, the day of the week could be determined for each modelled day, and the date on which certain events, such as non-pharmaceutical intervention (NPI) implementation or school reopening could be converted into the corresponding model day number.
5. An attribute transfer/selection sub-process – a newly infected (or seed infectious) individual's attributes from the synthetic population tables were transferred to active tracking variables for that person. The number of close contacts in each non-household setting (as described in detail below) were drawn from a multivariate lognormal distribution (mean values shown in Table 1 in the text) and assigned to the person. In first- and second-wave simulations, these contact numbers were proportionately lowered as shown in table 1 in the main text. Other attributes, such as whether the new infected person would develop either symptomatic or asymptomatic COVID-19 disease when they subsequently became infectious was sampled from a Bernoulli distribution as described below and stored as a tracking variable for the newly infected person. Likewise, whether infectious individuals would ultimately be detected was selected from one of two Bernoulli distributions, one for symptomatic cases and another for asymptomatic cases, according to the person's symptom status tracking variable. For adults of working age, whether they were considered to be essential workers was selected according to the method described below and the result, 1 for essential and 0 for not, was stored as a tracking variable for the person.
6. A school attendance sub-process (function) – used the model day number corresponding to dates of school reopening (in scenarios where schools did reopen), the day of the week, whether a particular student's classroom had been closed due to an outbreak (see below), and for urban high school students, whether the current week of the year was odd or even, to determine whether a given student or teacher would attend school on a particular model day (i.e. whether the tabular entries for schools and classrooms in the synthetic population would be accessible to active infectious children or teachers).
7. A workplace attendance sub-process (function) – used the model day number corresponding to the date of non-essential workplace closure, the day of the week, the employment status of an infectious individual, the essential worker status of the individual and also whether the individual had self-isolated or been quarantined to determine whether the infectious worker would have access to the tabular entries for workplaces in the synthetic population.
8. Transmission sub-processes (functions) for infectious individuals included those for household, college/university, neighborhood/district, city/region, school, and workplace transmission.
    a. The general features of these sub-processes (functions) – transmission functions would take as arguments the personal identifier of the transmitting infectious person, the number of close contacts selected for the person for the given setting, the probability of transmission per contact in that setting and the unique identifier of the setting. Using the latter, the function found the set of personal identifiers of the individuals associated with the particular setting. A for loop iterated from 1 to the number of close contacts and for each a member of the setting who remained susceptible was selected at random. Next, a random number in the range of 0 to 1 was drawn. If the random number was less than the probability of transmission,

© 2021 Naimark D et al. *JAMA Network Open.*

the selected person was infected and would enter the model as described above on the next day as an active agent starting in the infected state. The numbers of new infections per day were stored temporarily in matrices and then processed at the end of each simulation (see below).

b. The household transmission sub-process (function) – the setting was the transmitting persons household (which had a unique household identifier) and all members of the household were close contacts. The probability of transmission within the household depended on the age of the transmitting individual (as per the relevant rows in table 1 in the main text), whether the transmitting individual was symptomatic or asymptomatic, and the proportionate reduction in transmissibility due to the introduction of non-pharmaceutical interventions (NPIs) such as masking and physical distancing during the first and second wave of the COVID-19 pandemic as shown in table 1 in the main text.
c. The college/university sub-process (function) – the setting was the particular college or university that a student attended (which had a unique college/university identifier). The function was similar to the general description in section 'a', and the household function described above except that the number of close contacts per day at college or university was unknown and therefore selected through model calibration (table 1 in the main text), the members of the college or university consisted of the set of personal identifiers associated with the institution, and the probability of transmission between contacts was that of the 'Other settings row' modified by proportionate reductions due to NPIs as shown in Table 1 in the main text. The college/university sub-process had a minor role to play in the current iteration of the ABMCT because these institutions were modelled to be closed to in-person instruction after March 15, 2020. Nonetheless this sub-process was constructed so that the ABMCT could be used when post-secondary institutions reopen.
d. The neighborhood/district transmission sub-process (function) – the setting was a particular urban neighborhood or rural district (collectively referred to as patches with a unique patch identifier). The set of potential targets for an infectious individual was the set of people associated with the patch and the probability of transmission per contact was as per the 'Other settings' rows of and the proportionate reduction rows (table 1 in the main text).
e. The city of rural region transmission sub-process (function) – the setting was one of the 52 cities or the rural region (collectively referred to as regions with a unique region identifier). The set of potential targets for an infectious individual was the set of people associated with the region and the probability of transmission per contact was as per the 'Other settings' rows of and the proportionate reduction rows (table 1 in the main text). For college or university students attending their institution in person, the region identifier was changed to the one in which the institution was located.
f. The school transmission sub-process (function) – the setting was either a daycare facility or a classroom in a primary, elementary or high school (collectively referred to as classrooms each with a unique classroom identifier and, for primary, elementary and high schools, a unique school identifier). The set of potential targets for an infectious individual was the set of children associated with the

© 2021 Naimark D et al. *JAMA Network Open.*

classroom and the probability of transmission was that of the 'Other settings' for the first and second wave modified by the odds ratio for mask wearing versus not. The latter was assumed to be the estimate for the collective effort of schools to universally mask students and to keep them physically distant (table 1 of the main text).

g. The workplace transmission sub-process (function) – the setting was the infectious person's workplace (each of which had a unique workplace indentifier). The set of potential targets for an infectious individual was the set of coworkers associated with the particular workplace identifier and the probability of transmission was that of the 'Other settings' for the first and second wave (table 1 of the main text).

9. The case detection sub-process (function) – on each model day a proportion of symptomatic COVID-19 cases would be detected by their presentation to health care centres and a proportion of asymptomatic cases would be detected through contact tracing. These were modelled to be detected (confirmed) cases. This sub-process tallied the number of detected cases on each modelled day and stored them in temporary matrices.
10. The classroom closure sub-process (function) – at the beginning of each model day, this sub-process determined whether two or more infections had been detected (confirmed) among the students or the teacher in a classroom within the prior 14 days, and if so, closed the classroom. For currently closed classrooms, if the last infection was more than 14 days prior to the current model day, the classroom was reopened. The function also kept track of the number of daycare or classroom closures overall and for each classroom type: daycare, primary/elementary or high school and stored these values in temporary matrices.
11. The post-simulation accounting sub-process – at the end of the simulation, the data in temporary matrices for new, confirmed cases for each model day was compiled, multiplied by 14.5 to estimate case numbers on a Provincial scale and smoothed as described below. Similarly data on the overall number of classroom closures was compiled from matrices. These data were then exported as text files that could be used for summary tables and graphs.

**Assignment of symptomatic status**

The proportion of symptomatic COVID-19 cases by ten-year age category was obtained from Ontario's Case and Contact Management (CCM plus) database.[3] In order to calculate these proportions, missing symptomatic status in the CCM plus data was imputed by constructing a logistic regression equation employing data from individuals with known symptomatic status and with sex and age category as covariates (eTable2). The symptomatic status of an individual, i, with missing status in the database, was calculated as:

$$\ln(odds_i) = \beta_0 + \beta_1 X_{1i} + \cdots + \beta_n X_{ni}$$

$$odds_i = exp(\beta_0 + \beta_1 X_{1i} + \cdots + \beta_n X_{ni})$$

$$P_i = {odds_i}/{(1 + odds_i)}$$

$$S_i \sim Bernoulli(P_i).$$

© 2021 Naimark D et al. *JAMA Network Open.*

That is, the binary indicators for the person's age category and sex allowed calculation of the odds of being symptomatic which, in turn, allowed calculation the person's probability of being symptomatic, $P_i$. The individual's binary symptomatic status, $S_i = 1$ for symptomatic and $S_i = 0$ for non-symptomatic, was drawn from a Bernoulli distribution with a mean of $P_i$.

A similar method was used in the ABMCT model to assign symptomatic status to individuals infected with COVID-19 given the age and sex designation for an individual sampled along with that person's other characteristics from SPSD/M [1]. Since, the SPSD/M data source contains only male and female sex, the logistic regression equation was modified to fold the 'Other sex' effect into a revised intercept value:

$$\ln(odds) = \beta_0 + \beta_1\bar{X}_1 + \cdots + \beta_n\bar{X}_n$$
$$\beta_0 = \ln(odds) - \beta_1\bar{X}_1 - \cdots - \beta_n\bar{X}_n$$
$$\beta_0' = \beta_0 + \beta_n\bar{X}_n = \ln(odds) - \beta_1\bar{X}_1 - \cdots - \beta_{n-1}\bar{X}_{n-1}$$
$$\ln(odds) = \beta_0' + \beta_1\bar{X}_1 + \cdots + \beta_{n-1}\bar{X}_{n-1} ,$$

where $\bar{X}_n$ is the average of the covariate for 'Other sex' which, since the covariate is a categorical binary, equals the proportion of the CCM plus cohort with 'Other' as the listed sex.

**Modelled process of case detection (confirmation)**

Symptomatic individuals could present to an emergency department (ED) where they could either be admitted to hospital or sent home to isolate until recovered. Symptomatic individuals who did not visit an ED could self-isolate until recovery. A proportion of symptomatic cases would present for testing and be recorded as confirmed cases while a proportion of asymptomatic cases could be detected through contact tracing, reported as confirmed cases, and quarantined until recovery. The probability of detection, i.e. of an infectious individual being a confirmed case, during the first wave of COVID-19 was estimated to be 20% by Tuite et al[4]. in their compartmental transmission model. In addition, we estimated the probability of detection of an infectious individual in Ontario separately, for symptomatic and asymptomatic cases, via the cumulative number of confirmed cases until June 9, 2020, and the seroprevalence of anti-Covid19 antibodies reported by Public Health Ontario until that date of ~1%.[5] As a simplifying assumption, the proportions of symptomatic and asymptomatic cases reported in the CCM plus database in June, 2020, 0.79 and 0.21, respectively, were applied to the general population in the calculation of detection probabilities. For symptomatic individuals:

pDetect|symptomatic ~ cumulative cases/(0.79*population of Ontario*0.01) = 22394/(0.79*14.5M*0.01) = 0.195,

and for asymptomatic individuals:

pDetect|asymptomatic ~ 4244/(0.21*14.5M*0.01) = 0.139.

Given the uncertainty in these estimates, the probabilities of detection were refined via model calibration as described below.

The absorbing states in the ABMCT were 'recovered' or 'admitted to hospital' - where individuals could either die or recover. Death outside of hospital, such as in a long-term care

(LTC) facility, was not considered in the current iteration of the model nor was nosocomial transmission within hospitals.

**Modelled selection of the number of contacts of an infected individual**

Susceptible persons were modelled to be potentially infected via close contact with an infectious individual. Mixing of individuals was assumed to occur randomly within households, classrooms, college or university campuses, workplaces, neighborhoods or districts, and regions. The average number of close contacts per day, prior to the institution of public health restrictions in March, and April 2020, in the settings mentioned above was based on the CONNECT study [6] (Table 1 main text). For a given individual, the number of household contacts per day was assumed to be equal to the number of individuals in the household. The number of potential close contacts in non-household settings was obtained by rounding samples from a multivariate lognormal distribution to the nearest integer assuming a standard deviation of the log counts of 20% of the mean log count and correlation coefficients among settings of 0.9. This ensured that potential close contact numbers were always positive, that there was a rightward skew in the distribution of contact numbers such that a small proportion of individuals had very high potential daily contact numbers, and that a sampled daily close contact number in one setting was correlated with the number in other settings. The latter sampling provided the number of potential close contacts. The actual number of close contacts during a day could be constrained by the total number of individuals in a particular setting (in a workplace, classroom, etc.) (eFigures 2, 3, and 4).

## Calibration to Ontario's daily, new, symptomatic, confirmed case counts – first wave

For the first wave, the model was seeded with 150 infectious individuals who were selected from the synthetic population at random on February 22, 2020 (day 0). Each of the seed individual's number of days of prior infectiousness was drawn from a uniform distribution. There was a seven-day run-in period until February 29, 2020 when cases began to be reported by the model. As a model validation step, we calibrated the daily number of new, confirmed, symptomatic cases outside of LTC facilities reported by the model from February 29 until July 7, 2020, to the observed number of similarly defined cases in the CCM plus database.[3] We assumed an initial unmitigated rise in infections from the origin of the model on February 22, 2020, until March 7, 2020, at which time there was a modelled spontaneous reduction in close contact numbers, followed by closure of schools, colleges and universities on March 15, 2020, and, ultimately, closure of workplaces, with the exception of essential workers, which had an effect on April 8, 2020 (Table 1 in the main text).

The proportion of workers who were essential was estimated for each SPSD/M industry type as one minus the proportion of teleworkers in the particular industry derived from Statistics Canada data obtained on May 29, 2020[7] (eTable3). Assignment of an individual worker to essential status was by means of drawing from a Bernoulli distribution with a mean equal to the proportion of essential workers for that individual's SPSD/M industry designator.

© 2021 Naimark D et al. *JAMA Network Open.*

Calibration was achieved via a combination of manual search of the calibration parameters and via the bound optimization by quadratic approximation (BOBYQA) directed search algorithm [8] which seeks to minimize a goodness-of-fit statistic defined as the sum of squared differences between modeled and observed daily case counts. To ease the calibration process, observed and modelled case counts were subjected to Gaussian kernel smoothing:

$$\widetilde{c_t} = \frac{\sum_i^N K(t,i) \cdot c_i}{\sum_j^N (t,i)}$$

where a smoothed count, $\widetilde{c_t}$, at a particular time, t, is a weighted average of all of the case counts, $c_i$, from the first day to the Nth day, the last day in the dataset, with a Gaussian, kernel weighting function:

$$K(t,i) = exp\left(-\frac{(t-i)^2}{2b^2}\right)$$

where with ‘b’, is the bandwidth in days.

For first wave calibration, b = 7 days, provided an optimum of reduced stochasticity and a smoothed peak value close to the un-smoothed peak (eFigures 5 and 6).

Final model parameters of the calibrated model are presented in Table 1 in the main text, and calibrated model results for the number of daily, new, symptomatic, confirmed cases in Ontario from February 29 to July 7, 2020, are presented in eFigure 7.

**Calibration to Ontario’s daily new, confirmed case counts in September, 2020 – second wave**

For the COVID-19 second wave in Ontario, we obtained the cumulative number of confirmed cases, symptomatic and asymptomatic by ten-year age category from the CCM plus database. These counts were the inflated by dividing them by the case detection probabilities described above according to symptomatic status. The sum of these two counts was reduced to model scale and that number of individuals within the synthetic population were selected at random within the ten-year age categories to represent recovered and immune or deceased individuals as a result of COVID-19 infection in the first wave. The model was reseeded with randomly selected new infectious individuals, the number of whom was determined through model calibration. Contact numbers in settings outside households were adjusted to be lower than the pre-pandemic values according to the proportion of time that mobile telephones were outside of households in major Ontario cities in August, 2020, compared to the values prior to the pandemic.[9] After a run-in period from August 15 to 31, the model began reporting daily, new, confirmed, cases of COVID-19 on September 1, 2020. For calibration, the indicator for school re-opening was turned off, i.e. modelled schools remained closed, under the assumption that an effect of school opening in the observed case counts would take longer than two weeks to manifest. For Gaussian smoothing of observed daily case counts, a bandwidth of 2 days fit the data the best (eFigures 8 and 9). Using methods described above, we re-calibrated the model to the number of cases between September 1 and 30, 2020 (eFigure 10).

© 2021 Naimark D et al. *JAMA Network Open.*

**Calibration to Ontario's daily new, confirmed case counts from October 1 – 15, 2020**

For the first two weeks of October, there was an observed decrease in the rate of growth of new, confirmed COVID-19 cases to 0.8% per day in the CCM plus database. We replicated this reduction in growth by allowing modelled transmissions to occur randomly but limiting their number to 0.8% of the existing infectious individuals per day. For calibration, the indicator for school re-opening was turned on, i.e. modelled schools re-opened, under the assumption that an effect of school opening in mid-September would be reflected in the observed case counts in early October, 2020. Using methods described above, we re-calibrated the model to the number of cases between September 30 and October 15, 2020 (eFigures 11 to 13).

**Model assumptions**

Pre – pandemic close contact numbers were estimated for school, workplace, neighborhood/district and city/region derived from the CONNECT study.[6]

Close contact numbers in non-household locations was assumed to be correlated and follow a multivariate log-normal distribution with a standard deviation of 0.2 times the mean of the natural log contact numbers and a correlation coefficient among settings of 0.9.

All children aged 3 to 17 years were assumed to attend daycare facilities or schools.

Close contact numbers in non-household settings during the first wave of the pandemic were reduced by a factor determined through calibration (Table 1 in the main text).

All members of a household were modelled to be close contacts of an infected individual.

Younger children (< 10 years) transmit less effectively than older children (10 – 17 years) and adults.

A fixed, deterministic, latent period of 4 days for both asymptomatic and symptomatic individuals was assumed. On the fifth day after acquisition of infection, all individuals became infectious and able to transmit COVID-19 to others. Individuals destined to become symptomatic had a one-day pre-symptomatic period leading to a 5-day incubation period. A fixed, deterministic infectious period of 15 days was assumed.

Asymptomatic cases could be detected through contact tracing. In this iteration of the ABMCT, detection through random tracing was not considered.

The probability of detection for symptomatic and asymptomatic cases based on sero-prevalence data from Canadian Blood Services as described in the eMethods above.

The probability that symptomatic cases would self-isolate was estimated to be 90% within 3 days of symptom onset.

Recovery from COVID-19 was assumed to confer immunity to re-infection for the duration of the simulation.

Individuals admitted to hospital were considered to be permanently removed from the susceptible population because of either death in hospital or recovery and then discharge.

© 2021 Naimark D et al. *JAMA Network Open.*

Mortality due to COVID-19 in community settings such as long-term care settings was not modelled nor was nosocomial transmission within hospitals.

**Adherence to the CHEERS reporting guideline for modelling studies**

Item 1 – the title indicates that the work was a simulation-based modelling study.

Item 2 - the abstract provides a structured summary of objectives, setting, methods, results and conclusions

Item 3 – the introduction provides an explicit statement of the broader context for the study and presents the study question and its relevance for policy decisions.

Item 4 – The population characteristics of the synthetic population have been described in the main, text, eMethods and eTables 1 – 3.

Item 5 – relevant aspects of the system within which decisions have to be made have been provided in the description of the locations of COVID-19 transmission in the main text and eMethods.

Item 6 – the perspective of the study is not relevant since it is not a cost-utility analysis.

Item 7 – the interventions being compared are described in the methods section of the main text along with the rational for choosing them.

Item 8 – the time horizon of the analysis on October 31, 2020, is stated explicitly. Longer time-horizons would be inappropriate because of the rapid evolution of the pandemic.

Item 9 – discounting is not relevant because the time horizon is less than a year and time-in-state incremental utilities and costs are not a feature of the model.

Item 10 – the outcome used as the measure of benefit was the difference in incident and cumulative COVID-19 cases due to school re-opening versus keeping them closed. This is the most relevant benefit for the policy regarding school opening in the midst of a pandemic.

Item 11 – there is no single measure of effectiveness of an intervention. Rather, available estimates from multiple sources in the literature were subject to refinement via model calibration.

Item 12 – patient preferences are not relevant for the current study.

Item 13 – estimated resources consumed are not relevant for the current study.

Item 14 – unit costs and currency conversions are not relevant for this study.

Item 15 – there two most common decision analytic structures for transmission models include continuous time compartmental models and discrete time agent-based models. This study employed the latter structure to be able to model patient-level interactions and transmission mitigation strategies.

Item 16 – details regarding assumptions of the model are reported in the Methods section in the main text and in the ‘model assumptions’ section above.

© 2021 Naimark D et al. *JAMA Network Open.*

Item 17 – the current study described the method used to impute missing symptomatic status in the CCM plus database, no other methods were required for skewed or censored data or for pooling data. Since there were no time-in-state quantities calculated by the model, half-cycle or within-cycle correction was not required. Since the model was based on a scaled down version of the entire Ontario population, population heterogeneity and uncertainty was not considered.

© 2021 Naimark D et al. *JAMA Network Open.*

**eTable 1.** Comparison of Characteristics of the Synthetic Population Employed in Simulations and the Corresponding Values for the Ontario Population

| Characteristic | Synthetic population | Ontario population |
|---|---|---|
| **Age category** | | |
| 0 to1 | 1.92% | 1.89% |
| 2 to 3 | 2.14% | 2.15% |
| 4 to 17 | 14.61% | 14.56% |
| 18 to 34 | 22.58% | 22.62% |
| 35 to 64 | 40.13% | 40.05% |
| 65 and older | 18.62% | 18.73% |
| **Sex** | | |
| Female | 50.73% | 50.61% |
| Male | 49.27% | 49.39% |
| **Household size** | | |
| 1 | 29.01% | 29.14% |
| 2 | 33.12% | 32.96% |
| 3 | 15.16% | 15.25% |
| 4 | 15.44% | 15.41% |
| 5 | 5.06% | 5.02% |
| 6 | 1.65% | 1.66% |
| 7 | 0.54% | 0.55% |
| **Region** | | |
| Barrie | 1.10% | 1.09% |
| Belleville | 0.37% | 0.36% |
| Brampton | 6.35% | 6.31% |
| Brant | 0.26% | 0.26% |
| Brantford | 0.75% | 0.75% |
| Brockville | 0.61% | 0.64% |
| Burlington | 1.41% | 1.42% |
| Cambridge | 0.98% | 0.99% |
| Clarence-Rockland | 0.78% | 0.78% |

© 2021 Naimark D et al. *JAMA Network Open.*

| | | |
|---|---|---|
| Cornwall | 0.32% | 0.33% |
| Dryden | 0.25% | 0.24% |
| Elliot Lake | 0.32% | 0.31% |
| Greater Sudbury | 1.21% | 1.22% |
| Guelph | 1.06% | 1.05% |
| Haldimand County | 0.32% | 0.32% |
| Hamilton | 5.30% | 5.30% |
| Kawartha Lakes | 0.53% | 0.54% |
| Kenora | 0.46% | 0.46% |
| Kingston | 0.93% | 0.93% |
| Kitchener | 1.82% | 1.83% |
| London | 2.98% | 2.98% |
| Markham | 2.63% | 2.64% |
| Mississauga | 6.99% | 7.00% |
| Niagara Falls | 0.64% | 0.65% |
| Norfolk County | 0.46% | 0.45% |
| North Bay | 0.35% | 0.35% |
| Orillia | 0.22% | 0.22% |
| Oshawa | 1.24% | 1.26% |
| Ottawa | 9.40% | 9.41% |
| Owen Sound | 0.64% | 0.64% |
| Pembroke | 0.45% | 0.41% |
| Peterborough | 0.58% | 0.58% |
| Pickering | 0.66% | 0.66% |
| Port Colborne | 0.55% | 0.56% |
| Prince Edward County | 0.74% | 0.74% |
| Quinte West | 0.31% | 0.31% |
| Richmond Hill | 1.50% | 1.52% |
| Sarnia | 0.50% | 0.50% |
| Sault Ste. Marie | 0.51% | 0.50% |
| St. Catharines | 1.01% | 1.01% |

© 2021 Naimark D et al. *JAMA Network Open.*

| | | |
|---|---|---|
| St. Thomas | 0.28% | 0.28% |
| Stratford | 0.22% | 0.22% |
| Temiskaming Shores | 0.30% | 0.29% |
| Thorold | 0.60% | 0.60% |
| Thunder Bay | 0.80% | 0.81% |
| Timmins | 0.29% | 0.29% |
| Toronto | 27.31% | 27.24% |
| Vaughan | 2.39% | 2.40% |
| Waterloo | 0.82% | 0.83% |
| Welland | 0.37% | 0.38% |
| Windsor | 1.68% | 1.66% |
| Woodstock | 0.30% | 0.31% |
| Rural | 7.16% | 7.15% |
| **Industry** | | |
| Not applicable | 46.64% | 46.71% |
| Agriculture | 0.81% | 0.81% |
| Other primary | 0.37% | 0.38% |
| Utilities | 0.70% | 0.69% |
| Construction | 3.07% | 3.05% |
| Manufacturing | 5.68% | 5.72% |
| Trade | 7.91% | 7.91% |
| Transportation and warehousing | 2.66% | 2.64% |
| Finance, insurance, rea lestate and leasing | 3.60% | 3.60% |
| Professional, scientific and technical services | 4.35% | 4.36% |
| Management, administration, and other support | 2.51% | 2.48% |
| Educational services | 4.31% | 4.34% |
| Health care and social assistance | 6.03% | 5.97% |
| Information, culture, and recreation | 3.04% | 3.05% |
| Accommodation and food services | 3.28% | 3.26% |
| Other services | 2.06% | 2.09% |
| Public administration | 2.98% | 2.96% |

© 2021 Naimark D et al. *JAMA Network Open.*

**eTable 2.** Logistic Coefficients for Imputation of Missing Symptomatic Status and for Assignment of Symptomatic Status of Individuals Infected With SARS-CoV-2 in the Agent Based Model of COVID-19 Transmission (ABMCT)

| **Factor** | **Beta coefficient** | **SE** | **p-value** |
|---|---|---|---|
| Intercept | -0.70182 | 0.075148 | <0.001 |
| Age group 10 to 19 | -0.47205 | 0.091769 | <0.001 |
| Age group 20 to 29 | -0.76417 | 0.080276 | <0.001 |
| Age group 30 to 39 | -0.86852 | 0.081766 | <0.001 |
| Age group 40 to 49 | -0.92607 | 0.082403 | <0.001 |
| Age group 50 to 59 | -1.02056 | 0.082055 | <0.001 |
| Age group 60 to 69 | -0.77826 | 0.084047 | <0.001 |
| Age group 70 to 79 | -0.60985 | 0.088948 | <0.001 |
| Age group 80 or older | -0.10229 | 0.080993 | 0.207 |
| Male sex | 0.067427 | 0.026434 | 0.011 |
| Other sex[a] | 1.309702 | 0.145335 | <0.001 |

Data on symptomatic status was obtained from the Case and Contact Management (CCM plus) database for COVID-19 cases in Ontario.[3] For individuals with missing symptomatic status, we developed a logistic regression model, with coefficients listed in the table in order to perform imputation as described in the eMethods. SE – standard errors of the logistic beta coefficients.

[a] 'Other sex' refers to individuals who neither identify as male or female.

© 2021 Naimark D et al. *JAMA Network Open.*

**eTable 3.** Percentage of Essential Workers by Social Policy Simulation Database and Model (SPSD/M) Industry Designation

| SPSD/M Industry Type | SPSD/M industry designation | Percentage of essential workers |
|---|---|---|
| Not applicable | 0 | 43.2% |
| Agriculture | 1 | 61.4% |
| Other primary | 2 | 43.2% |
| Utilities | 3 | 43.2% |
| Construction | 4 | 60.9% |
| Manufacturing | 5 | 54.6% |
| Trade | 6 | 50.9% |
| Transportation and warehousing | 7 | 42.7% |
| Finance, insurance, real estate, and leasing | 8 | 35.9% |
| Professional, scientific, and technical services | 9 | 14.1% |
| Management, administration, and other support | 10 | 49.8% |
| Educational services | 11 | 33.7% |
| Health care and social assistance | 12 | 41.1% |
| Information, culture, and recreation | 13 | 31.4% |
| Accommodation and food services | 14 | 65.1% |
| Other services | 15 | 45.2% |

The proportion of workers who were essential was estimated for each SPSD/M industry type as one minus the proportion of teleworkers in the particular industry derived from Statistics Canada data obtained on May 29, 2020[7]. For ABMCT sampling purposes, the percentages were converted to proportions so that an individual simulated worker's essential status could be sampled from a Bernoulli distribution as described in the eMethods.

© 2021 Naimark D et al. *JAMA Network Open.*

**eTable 4.** Summary of Simulation Scenarios

| | | Nonpharmaceutical intervention scenarios | | |
|---|---|---|---|---|
| | | **1: No NPIs implemented on October 1, 2020** | **2: NPIs implemented on October 1, 2020** | **3: Replication of case counts, October 1- 15, 2020** |
| **School re-opening scenarios** | **A. Schools remained closed** | 1A | 2A | 3A |
| | **B. Schools re-opened on September 15, 2020** | 1B | 2B | 3B |

Schools remained closed – daycare facilities and primary, elementary and high schools remained closed on Sept 15, 2020; Schools re-opened on Sept 15, 2020: daycare facilities and primary, elementary and high schools re-opened on that date with safety measures as indicated in the text; no restrictions: the rise in daily new, confirmed, COVID-19 case counts observed between September 1 – 30, 2020, persisted until October 31, 2020; restrictions – on October 1, 2020, workplaces were closed except for essential workers, the population remained at home except for essential outings thereby reducing non-household contacts by 40% and probability of transmission of COVID-19 between contacts by 50% compared to the August, 2020, baseline; replication of case counts: a rise in daily new, confirmed, COVID-19 case counts observed between September 1 – 30, 2020, followed by a reduction in the rate of new case counts to 0.8% per day as observed in the first two weeks of October, 2020. For all public health scenarios, simulation data was reported by the model from September 1 to October 31, 2020.

© 2021 Naimark D et al. *JAMA Network Open.*

**eTable 5.** Summary of Main Simulation Results

| Non-pharmaceutical interventions | School status | Scenario | Estimated, new, confirmed, cases on Oct. 31, 2020 | Estimated, cumulative, confirmed, cases by Oct. 31, 2020 |
|---|---|---|---|---|
| | | | | |
| No NPIs implemented | Remain closed | 1A | 4414 (95% CrI: 3,491; 5,382) | 82372 (95% CrI:64448 ;102518) |
| | Reopened | 1B | 4740 (95% CrI 3,863; 5,691) | 86507 (95% CrI:68624 ;105505) |
| | Difference | (1B - 1A) | 326 (95% CrI: 196; 456) | 4135 (95% CrI: 4044; 4227) |
| | | | | |
| NPIs implemented | Remain closed | 2A | 714 (95% CrI: 568; 908) | 45112 (95% CrI:35873 ;56790) |
| | Reopened | 2B | 780 (95% CrI 580; 993) | 45058 (95% CrI:34781 ;55203) |
| | Difference | (2B - 2A) | 66 (95% CrI: 40; 92) | -54 (95% CrI: -123; 14) |
| | | | | |
| Replication of October 1 to 15, 2020 case counts[a] | Remain closed | 3A | 777 (95% CrI: 621; 993) | 34911 (95% CrI:27302 ;44127) |
| | Reopened | 3B | 803 (95% CrI 617; 990) | 35581 (95% CrI:28278;43986) |
| | Difference | (3B - 3A) | 26 (95% CrI: 0; 52) | 670 (95% CrI: 609; 732) |

Mean estimated new (incident), confirmed, cases on October 31, 2020, and estimated cumulative cases from September 1, 2020, until October 31, 2020, across 100 model repetitions as reported in the results section of the main text. Schools remained closed – daycare facilities and primary, elementary and high schools remained closed on Sept 15, 2020; Schools re-opened on Sept 15, 2020: daycare facilities and primary, elementary and high schools re-opened on that date with safety measures as indicated in the main text; no non-

© 2021 Naimark D et al. *JAMA Network Open.*

pharmaceutical interventions (NPIs): the rise in daily new, confirmed, COVID-19 case counts observed between September 1 – 30, 2020, persisted until October 31, 2020; NPIs imposed – on October 1, 2020, workplaces were closed except for essential workers, the population remained at home except for essential outings thereby reducing non-household contacts by 40% and probability of transmission of COVID-19 between contacts by 50% compared to the August, 2020, baseline; replication of case counts: a rise in daily new, confirmed, COVID-19 case counts observed between September 1 – 30, 2020, followed by a reduction in the rate of new case counts to 0.8% per day as observed in the first two weeks of October, 2020.
CrI – credible interval.
[a]The replication of the reduction in case counts in early October is not based on a particular non-pharmaceutical intervention but rather a cap on the growth of new cases of 0.8% per day.

© 2021 Naimark D et al. *JAMA Network Open.*

**eFigure 1.** Agent-Based Model of COVID-19 Transmission (ABMCT) Model Schematic

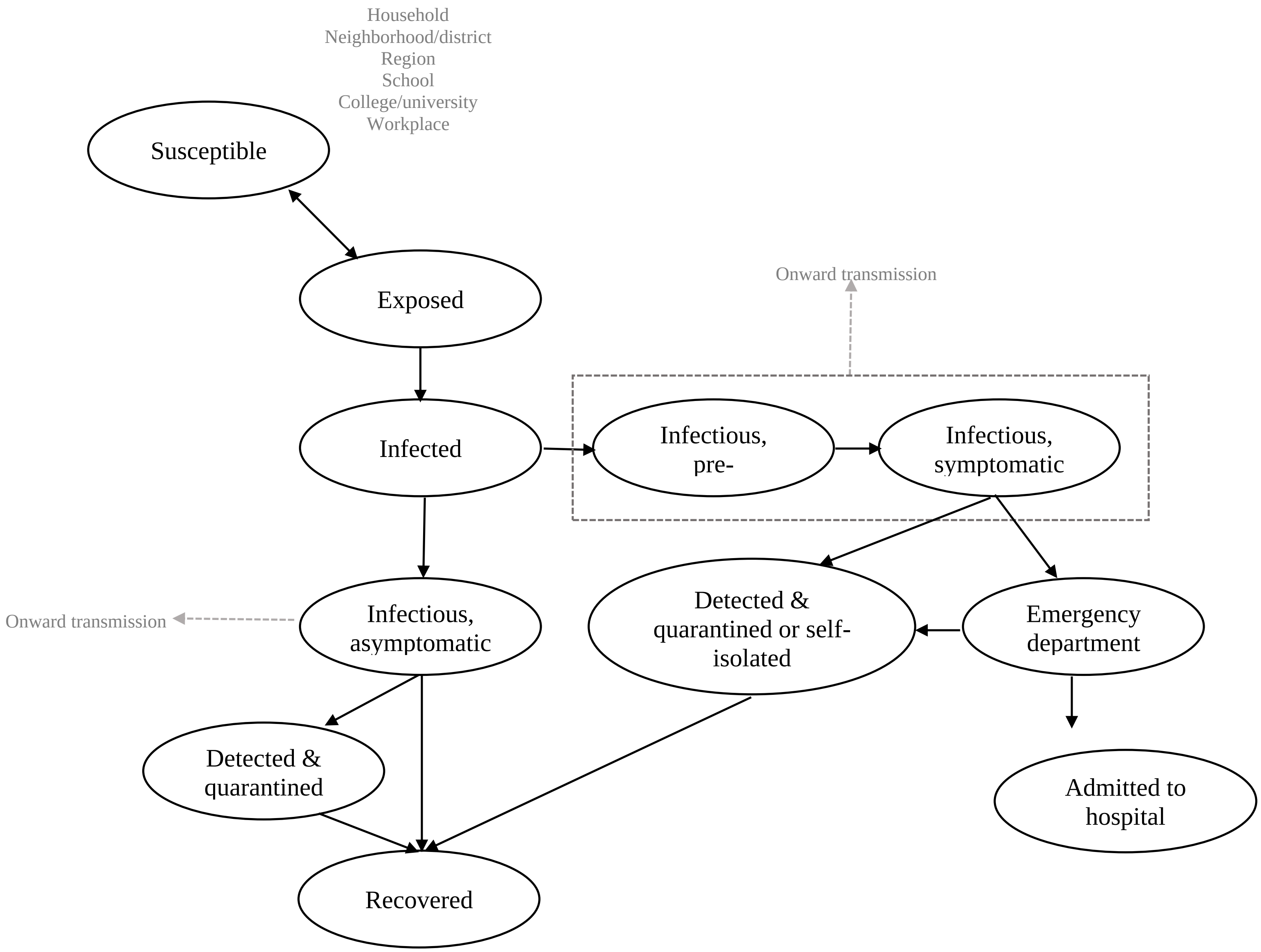


© 2021 Naimark D et al. *JAMA Network Open.*

**eFigure 2.** Demonstration of 5000 Samples From the Multivariate Lognormal Distribution of Daily Contacts at Work

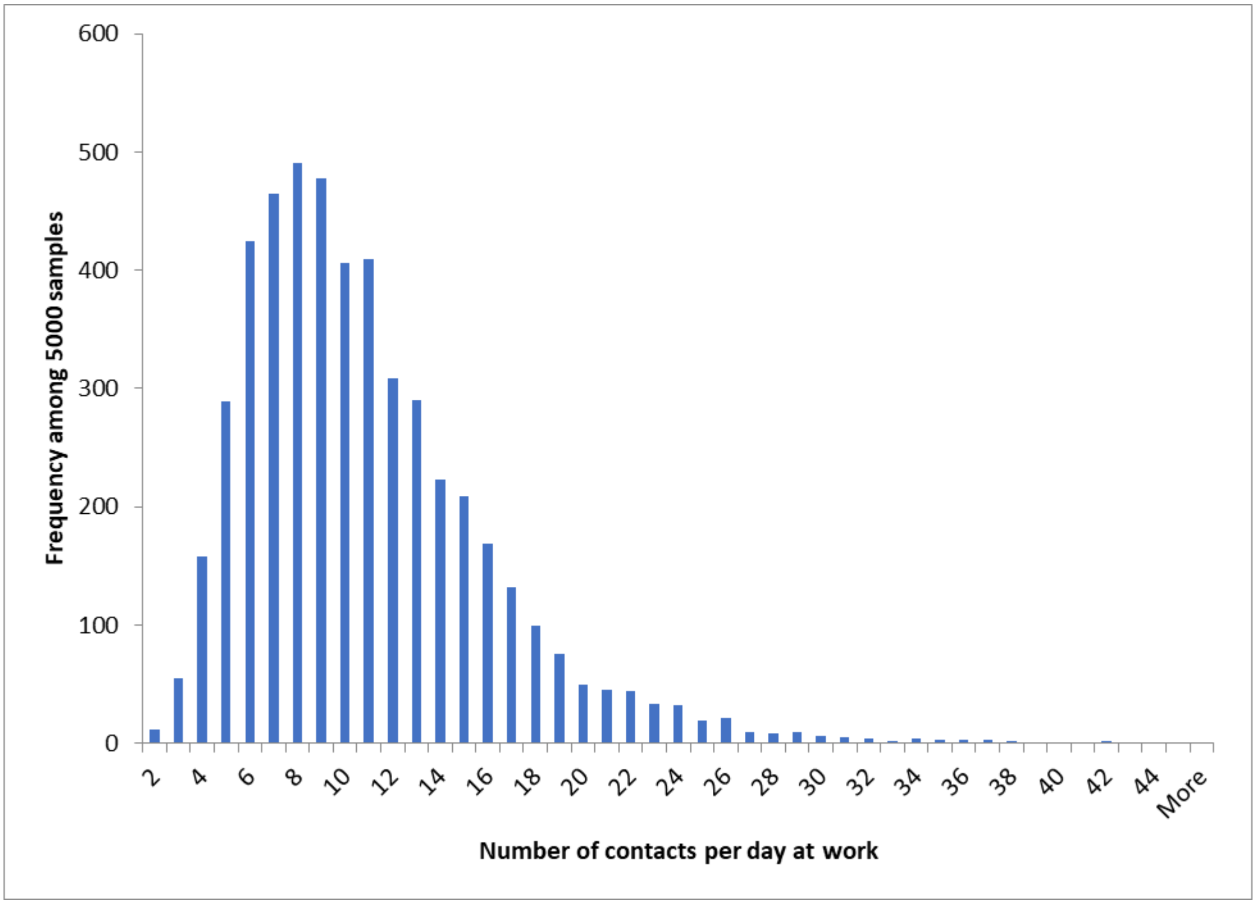


An individual sample produced the natural logarithm of work contacts which was then exponentiated and rounded to the nearest integer. The actual number of estimated daily contacts may be lower than the sampled number of potential daily contacts in smaller workplaces with fewer numbers of workers.

© 2021 Naimark D et al. *JAMA Network Open.*

**eFigure 3.** Demonstration of 5000 Samples From the Multivariate Lognormal Distribution of Potential Numbers of Daily Contacts at Day Care and Primary, Elementary, and High Schools

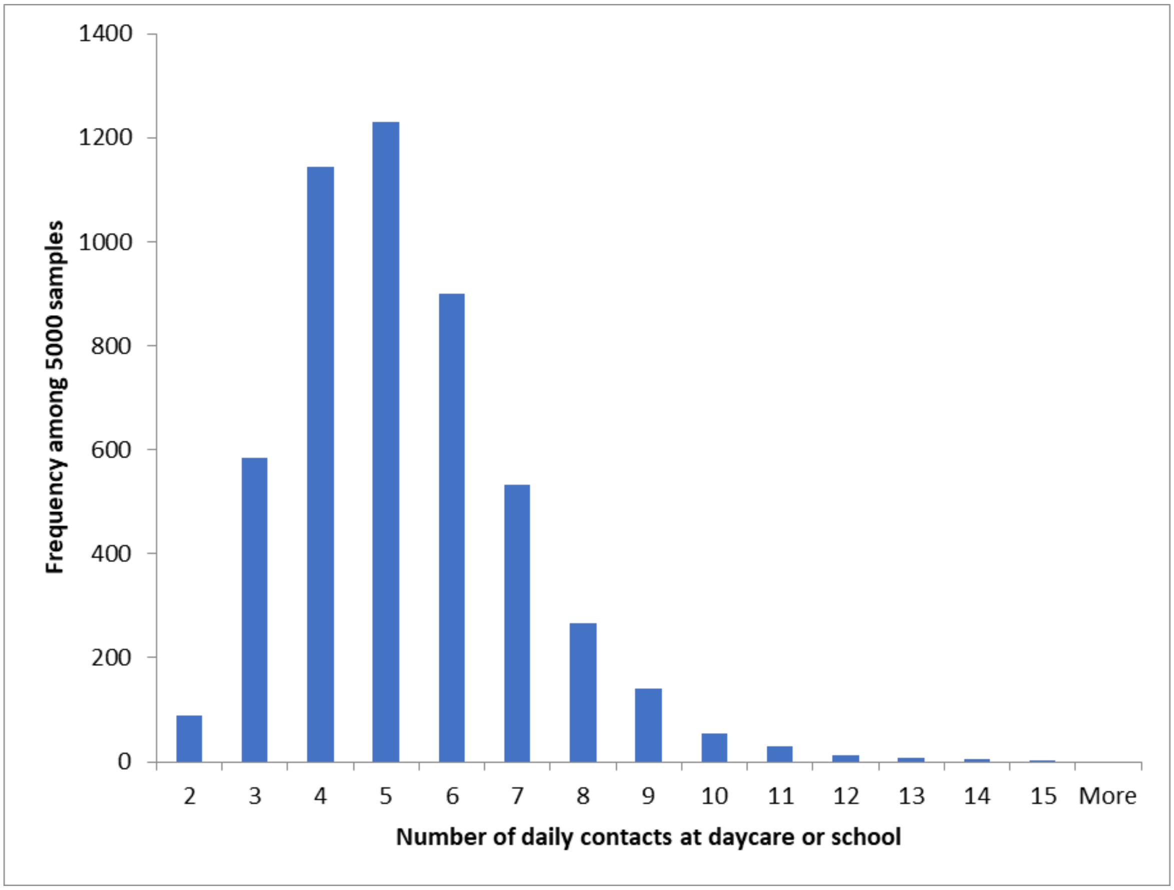


An individual sample produced the natural logarithm of contacts which was then exponentiated and rounded to the nearest integer. The actual number of daily contacts may be lower than the sampled number of potential daily contacts because of caps on daycare facility and schoolroom capacity.

© 2021 Naimark D et al. *JAMA Network Open.*

**eFigure 4.** Demonstration of the Correlation of 5000 Samples From the Multivariate Lognormal Distribution of Potential Numbers of Daily Contacts at Work and Within the Urban City or Rural Region

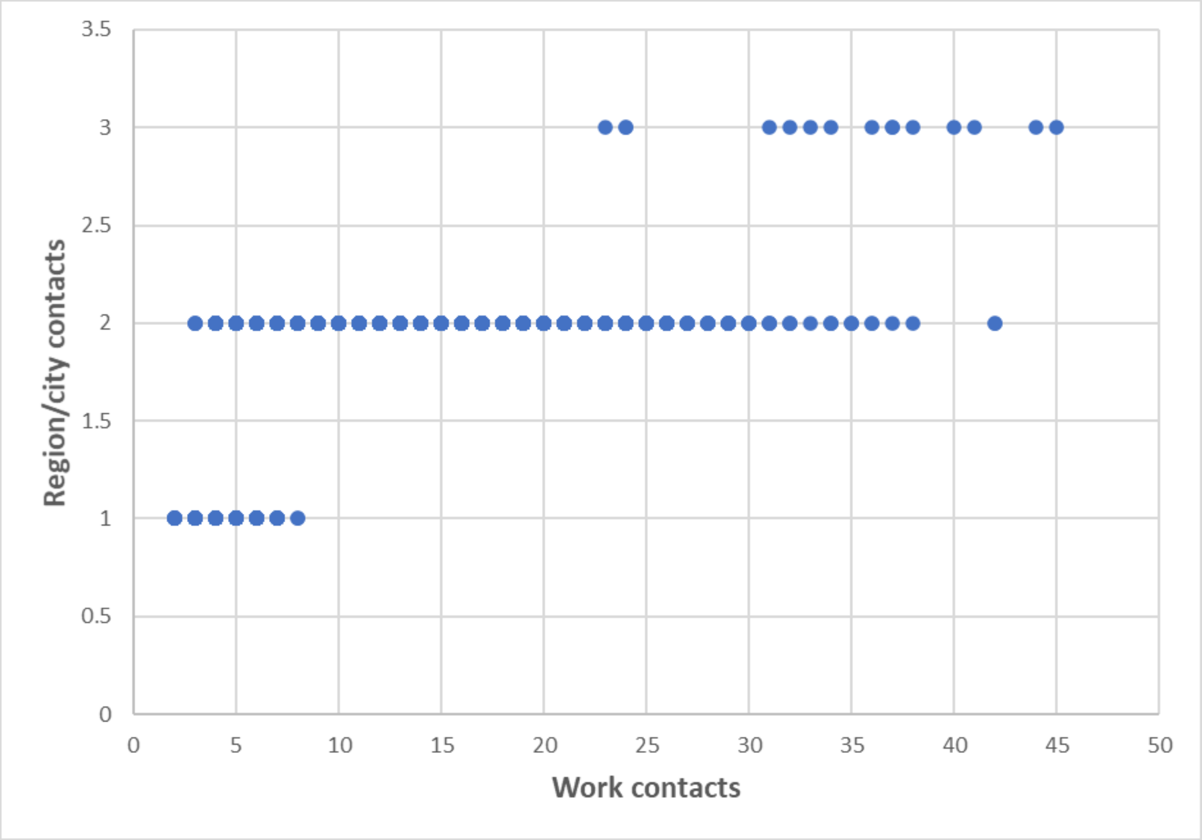


Note that each point on the graph represents more than one sample.

© 2021 Naimark D et al. *JAMA Network Open.*

**eFigure 5.** Observed vs Smoothed Daily New Symptomatic COVID-19 Case Counts (First Wave) for Various Gaussian Kernel Smoothing Bandwidths

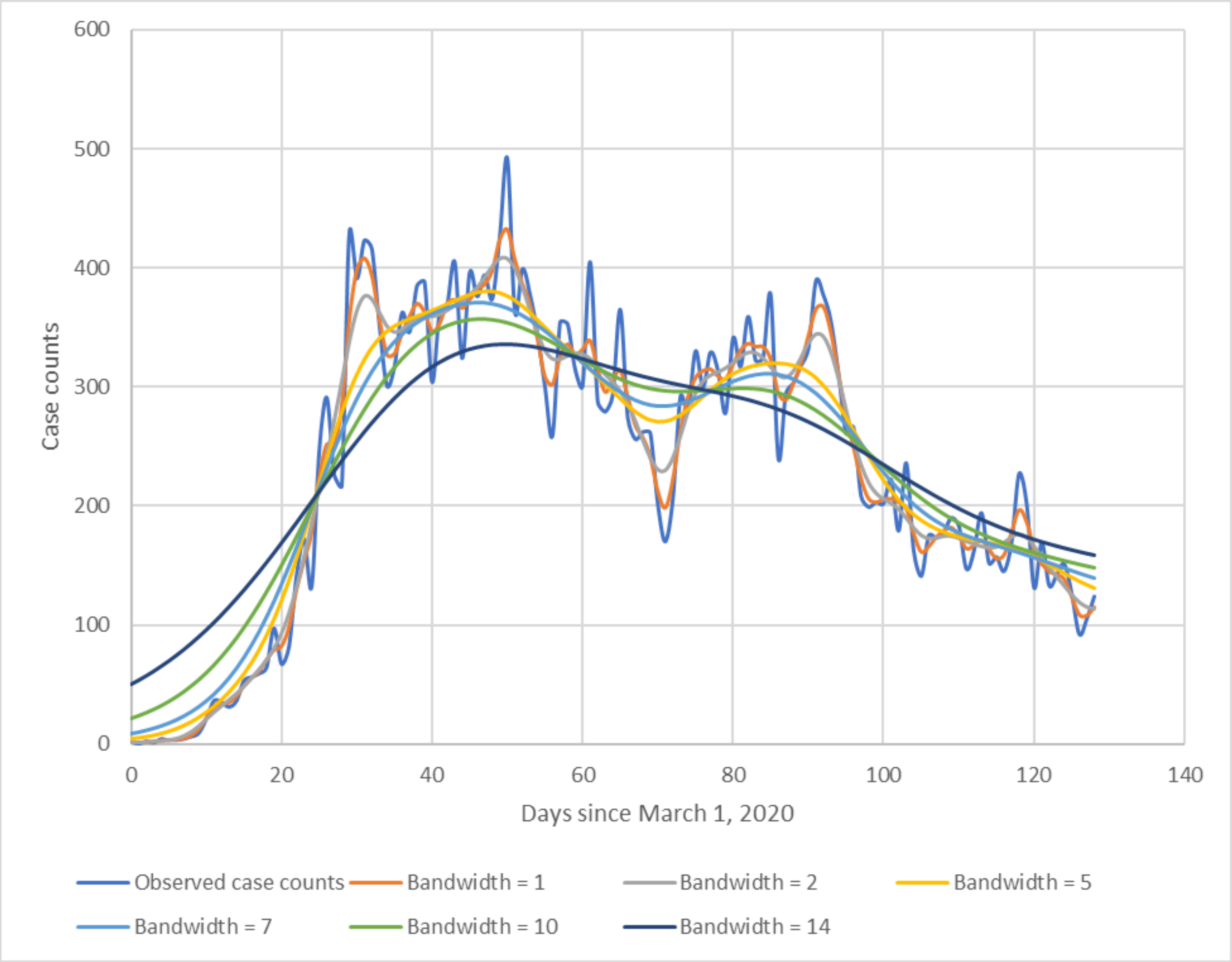


© 2021 Naimark D et al. *JAMA Network Open.*

**eFigure 6.** Observed vs Smoothed Daily New Symptomatic COVID-19 Case Counts (First Wave) for the Selected Gaussian Kernel Smoothing Bandwidth of 7 Days

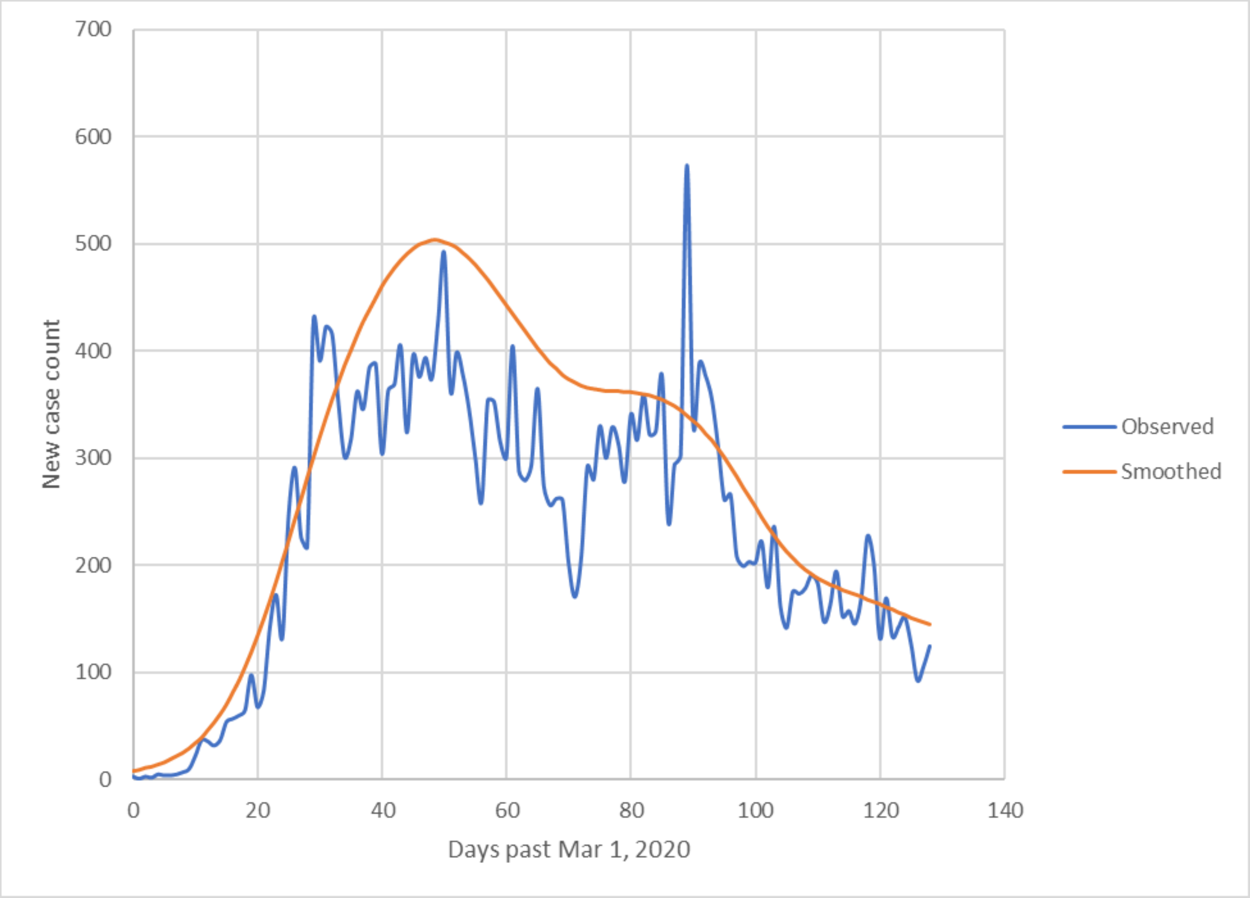


© 2021 Naimark D et al. *JAMA Network Open.*

**eFigure 7.** Model Calibration to Daily New Symptomatic Confirmed COVID-19 Cases in Ontario (First Wave)

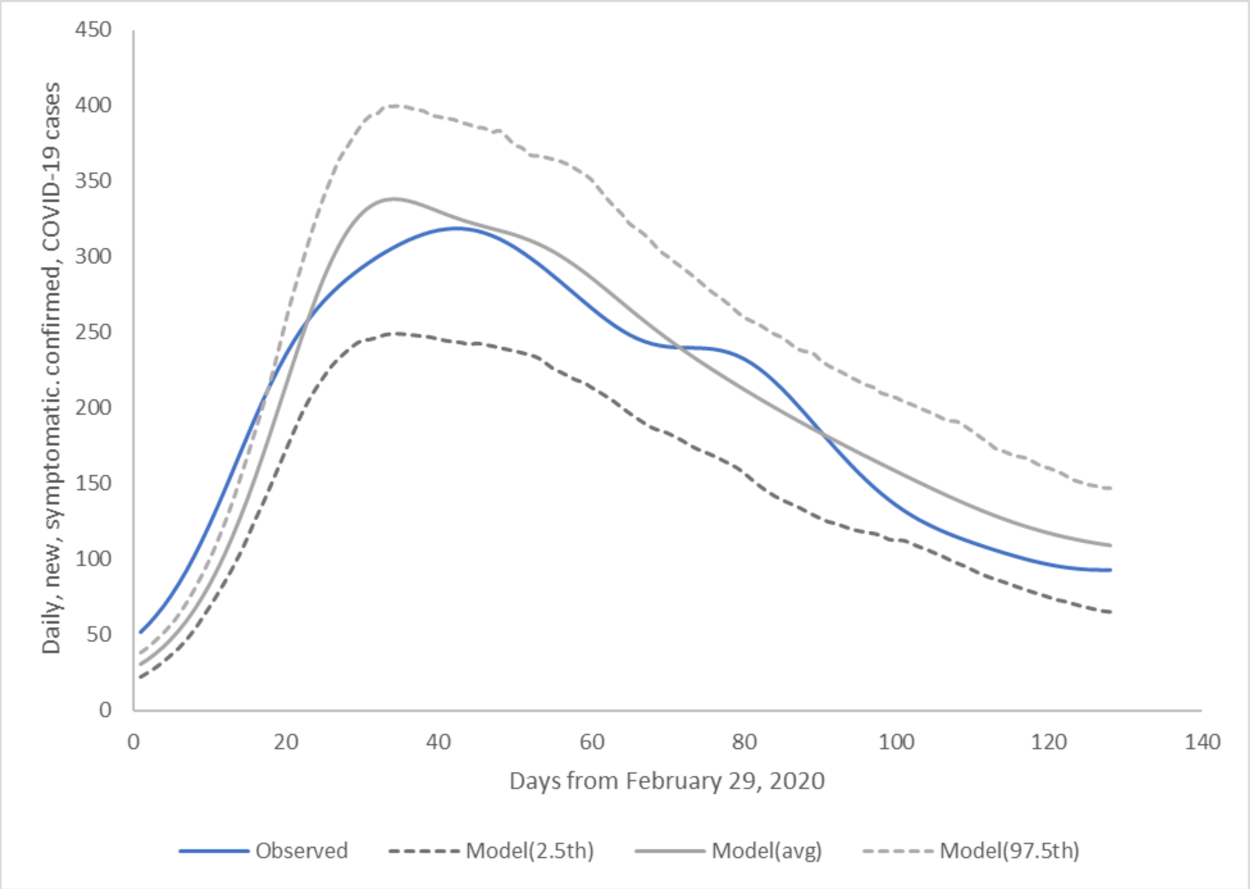


Observed cases in the CCM plus database (blue) compared to the average among 250 model repetitions (solid grey) along with the associated 2.5th and 97.5th percentiles (dotted grey) between February 29 (day 0) and July 7, 2020. Note, both observed and modelled counts were subjected to Gaussian kernel smoothing with a bandwidth of 7 days.

© 2021 Naimark D et al. *JAMA Network Open.*

**eFigure 8.** Observed vs Smoothed Daily New Symptomatic COVID-19 Case Counts (Second Wave) for Various Gaussian Kernel Smoothing Bandwidths

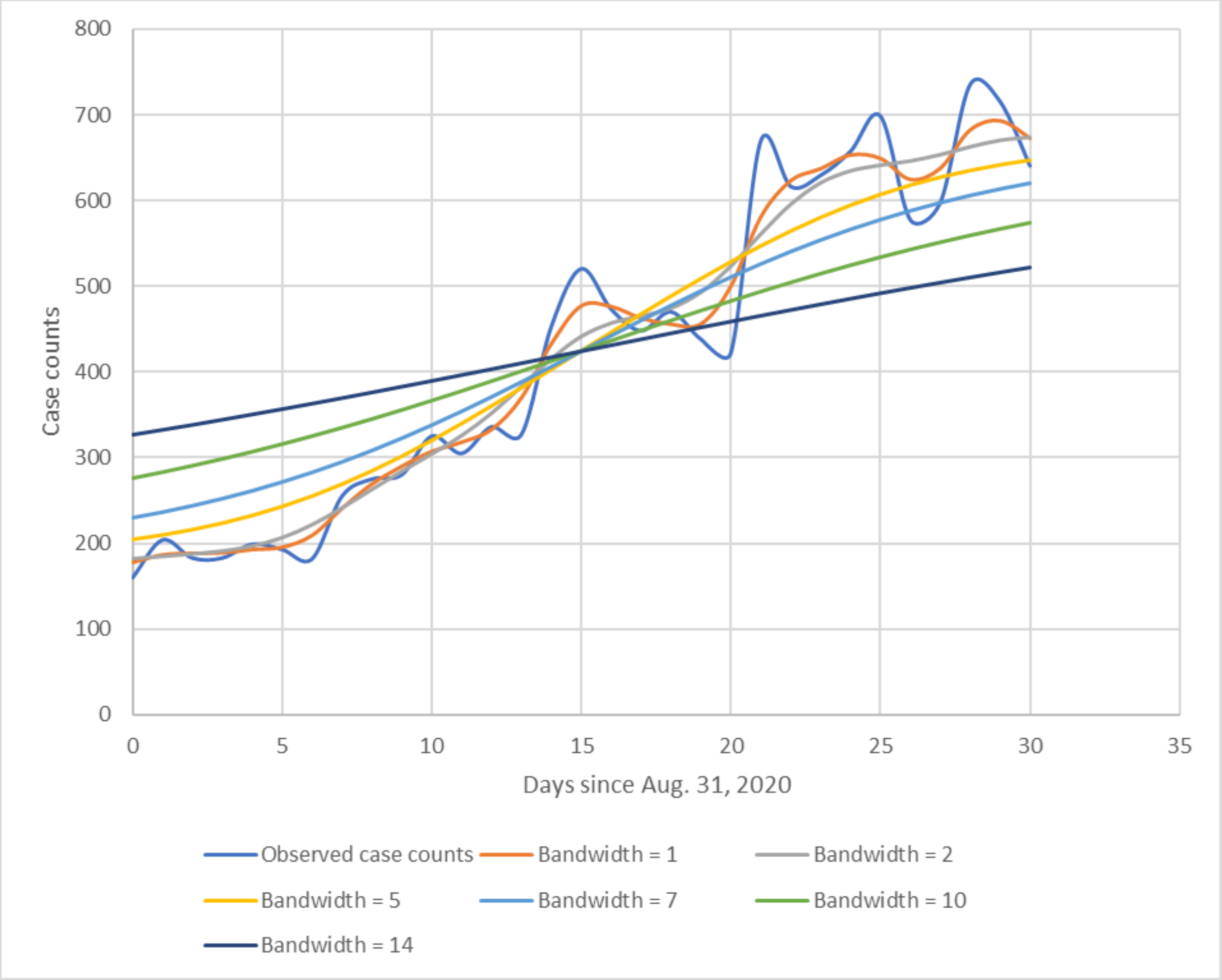


© 2021 Naimark D et al. *JAMA Network Open.*

**eFigure 9.** Observed vs Smoothed Daily New Symptomatic COVID-19 Case Counts (Second Wave) for the Selected Gaussian Kernel Smoothing Bandwidth of 2 Days

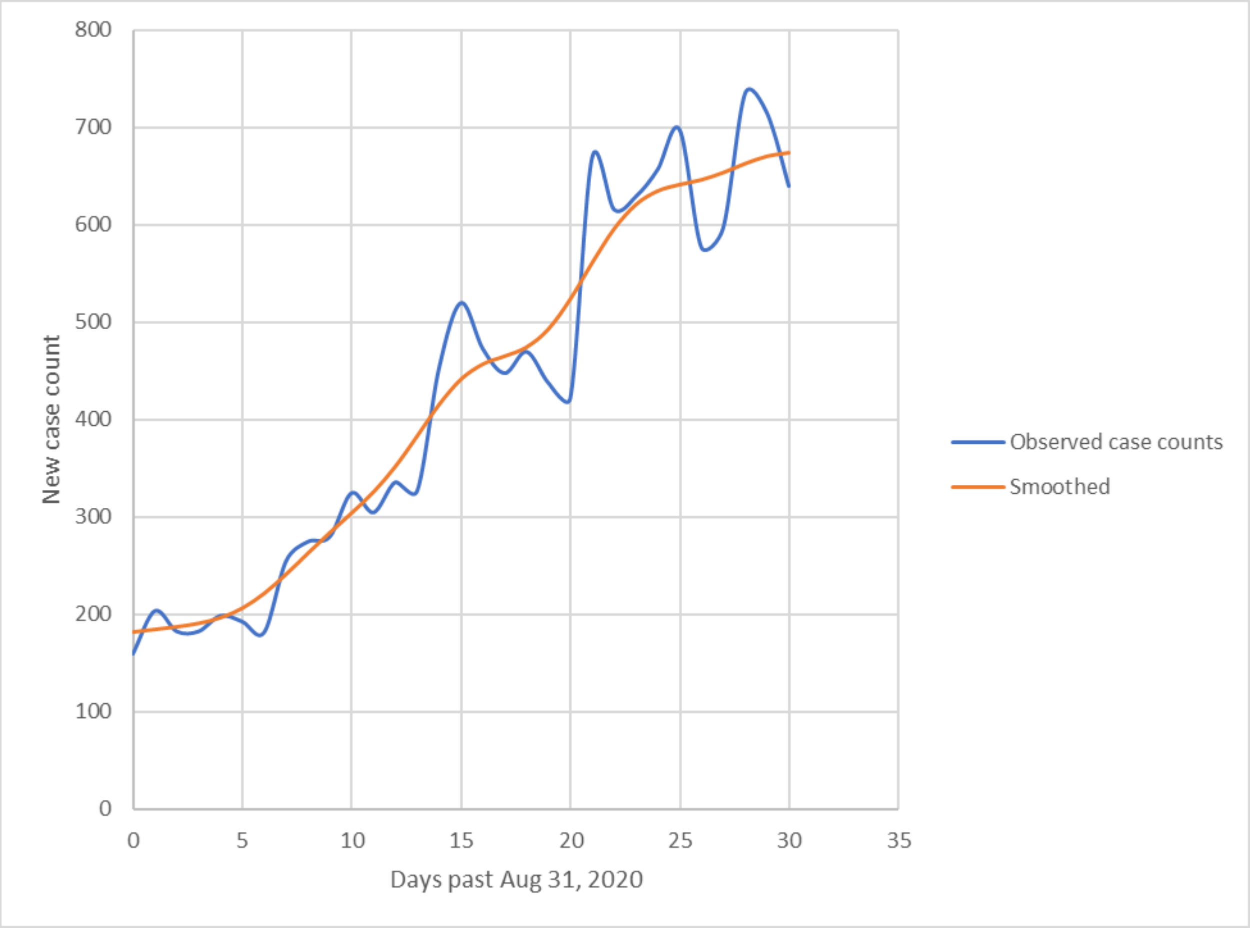


© 2021 Naimark D et al. *JAMA Network Open.*

**eFigure 10.** Model Calibration to Daily New Total Confirmed COVID-19 Cases in Ontario (Second Wave)

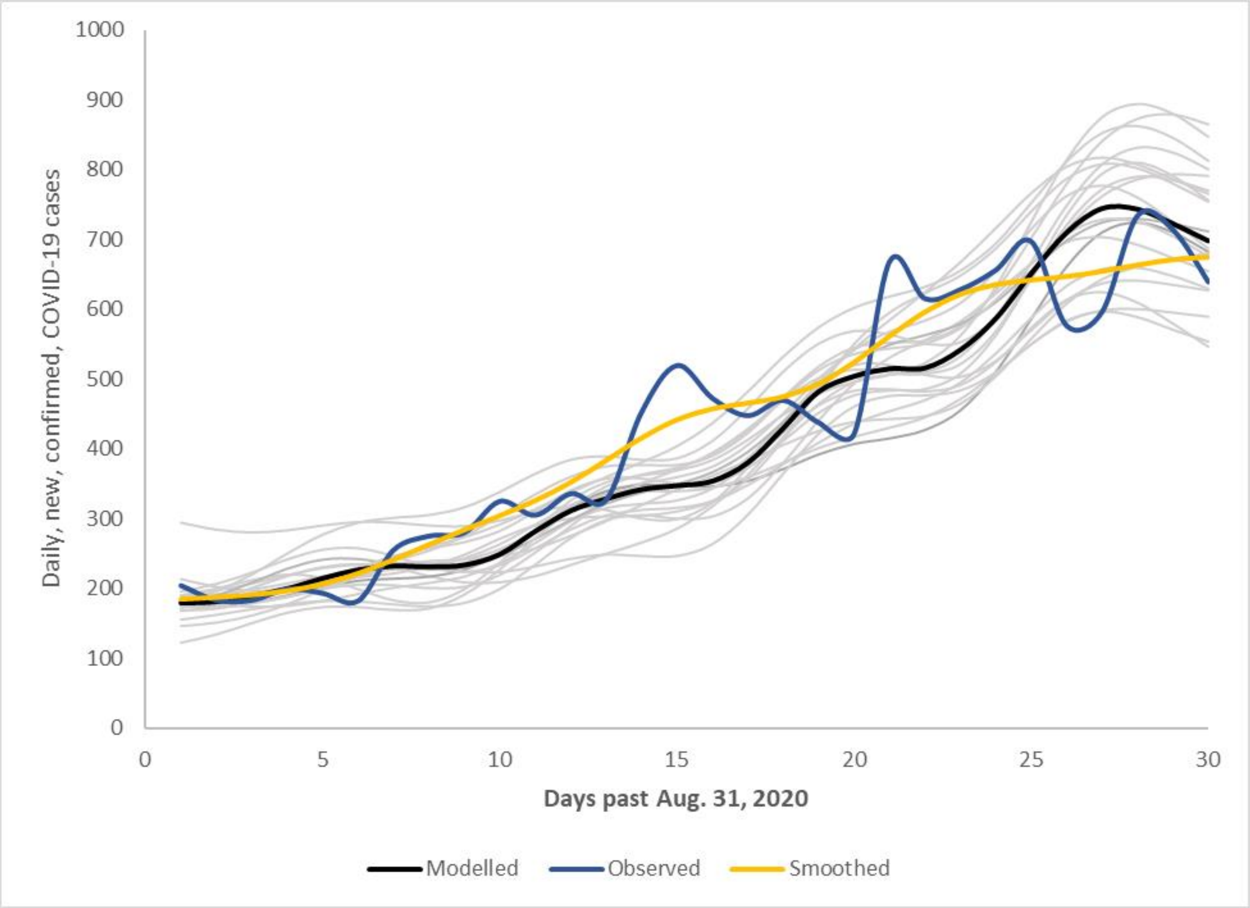


Observed cases in the CCM plus database (blue), after Gaussian kernel smoothing with a bandwidth of 2 days (gold), compared to the average among 20 model repitions (black) and to the individual calibrated replicates (grey) along between September 1 and 30, 2020. Note, both observed and modelled counts subjected to Gaussian kernel smoothing with a bandwidth of 2 days.

© 2021 Naimark D et al. *JAMA Network Open.*

**eFigure 11.** Observed vs Smoothed Daily New Symptomatic COVID-19 Case Counts During the First 15 Days of October 2020 for Various Gaussian Kernel Smoothing Bandwidths

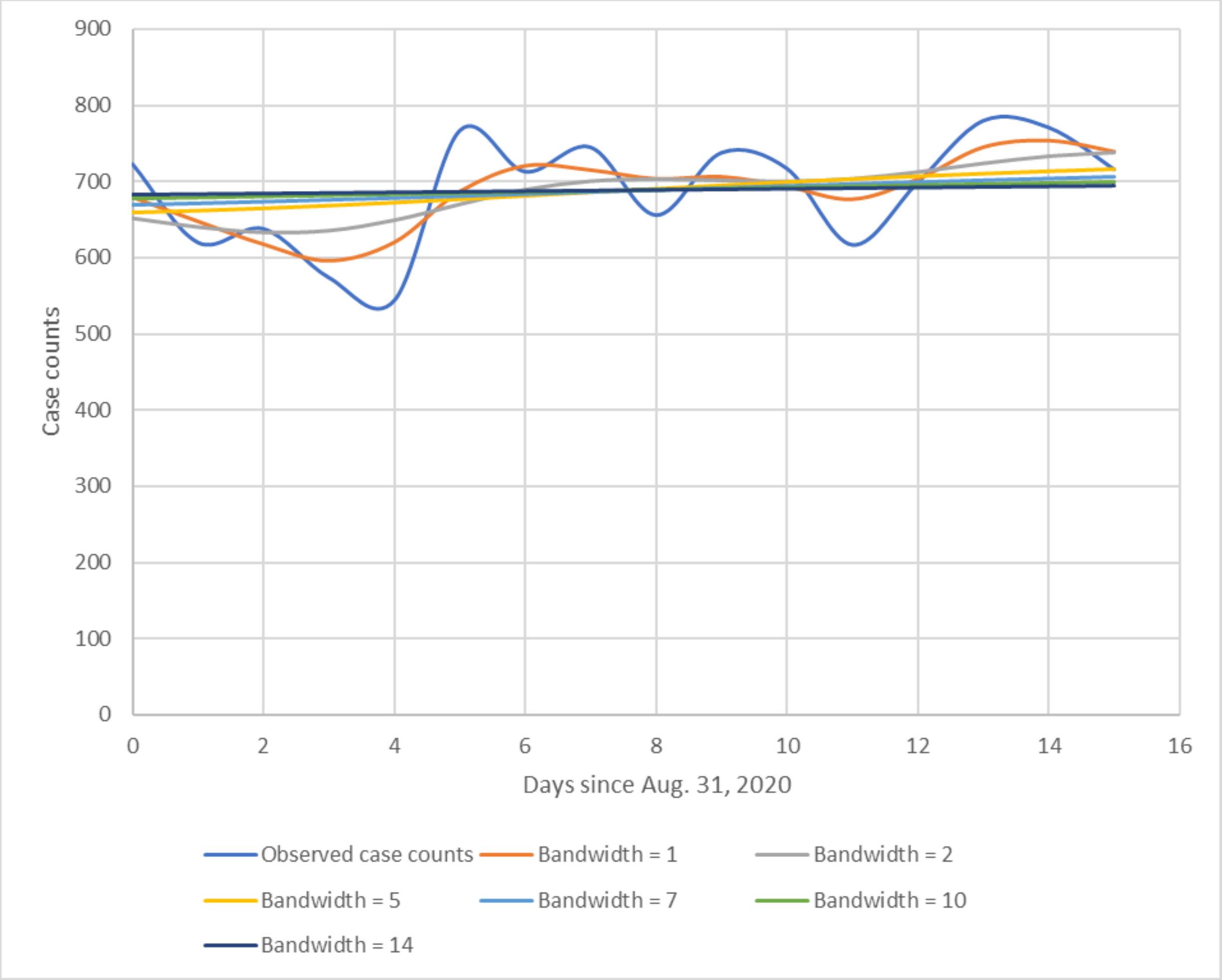


© 2021 Naimark D et al. *JAMA Network Open.*

**eFigure 12.** Observed vs Smoothed Daily New Symptomatic COVID-19 Case Counts During the First 15 Days of October 2020 for the Selected Gaussian Kernel Smoothing Bandwidth of 2 Days

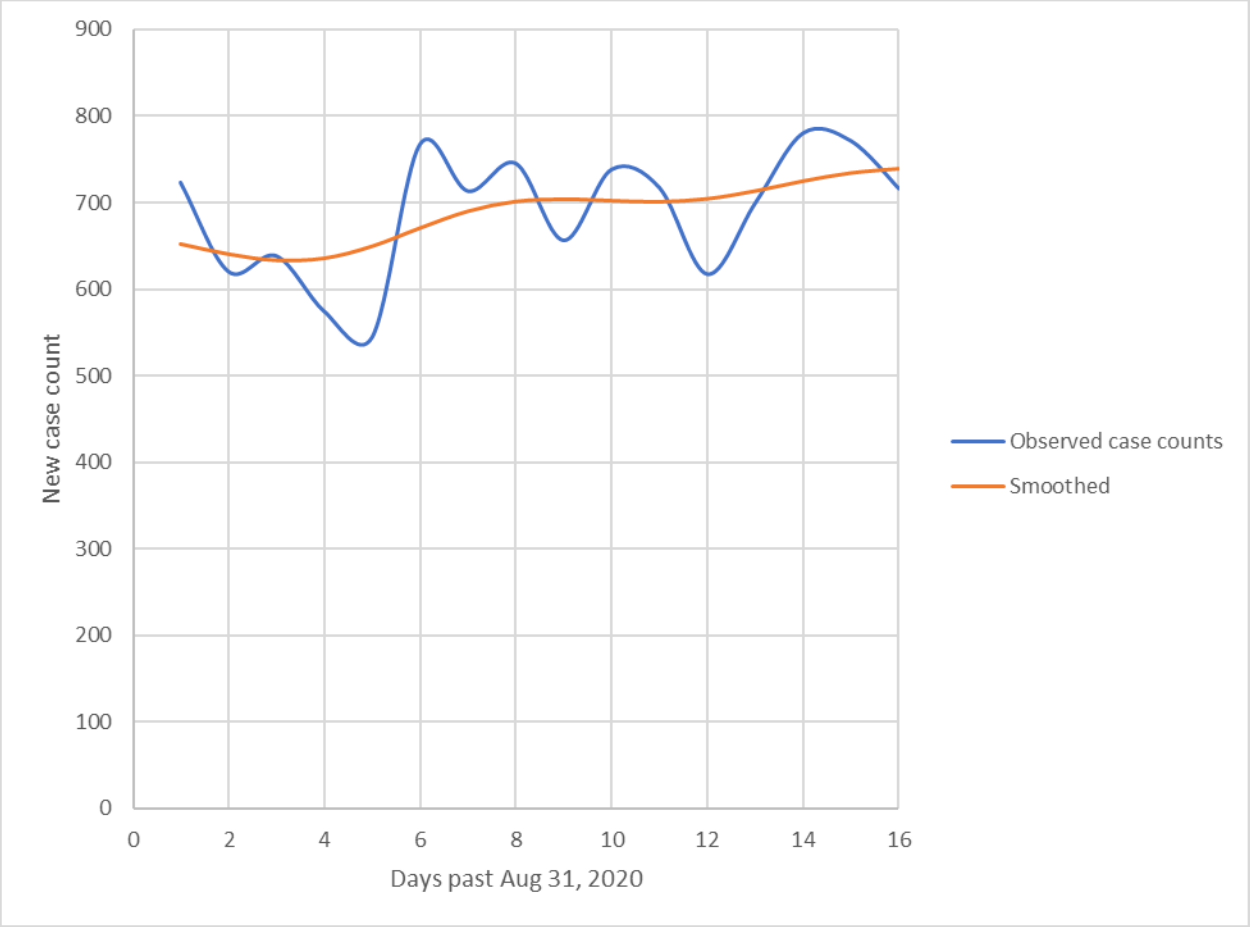


© 2021 Naimark D et al. *JAMA Network Open.*

**eFigure 13.** Model Calibration to Daily New Total Confirmed COVID-19 Cases in Ontario During the First 15 Days of October 2020

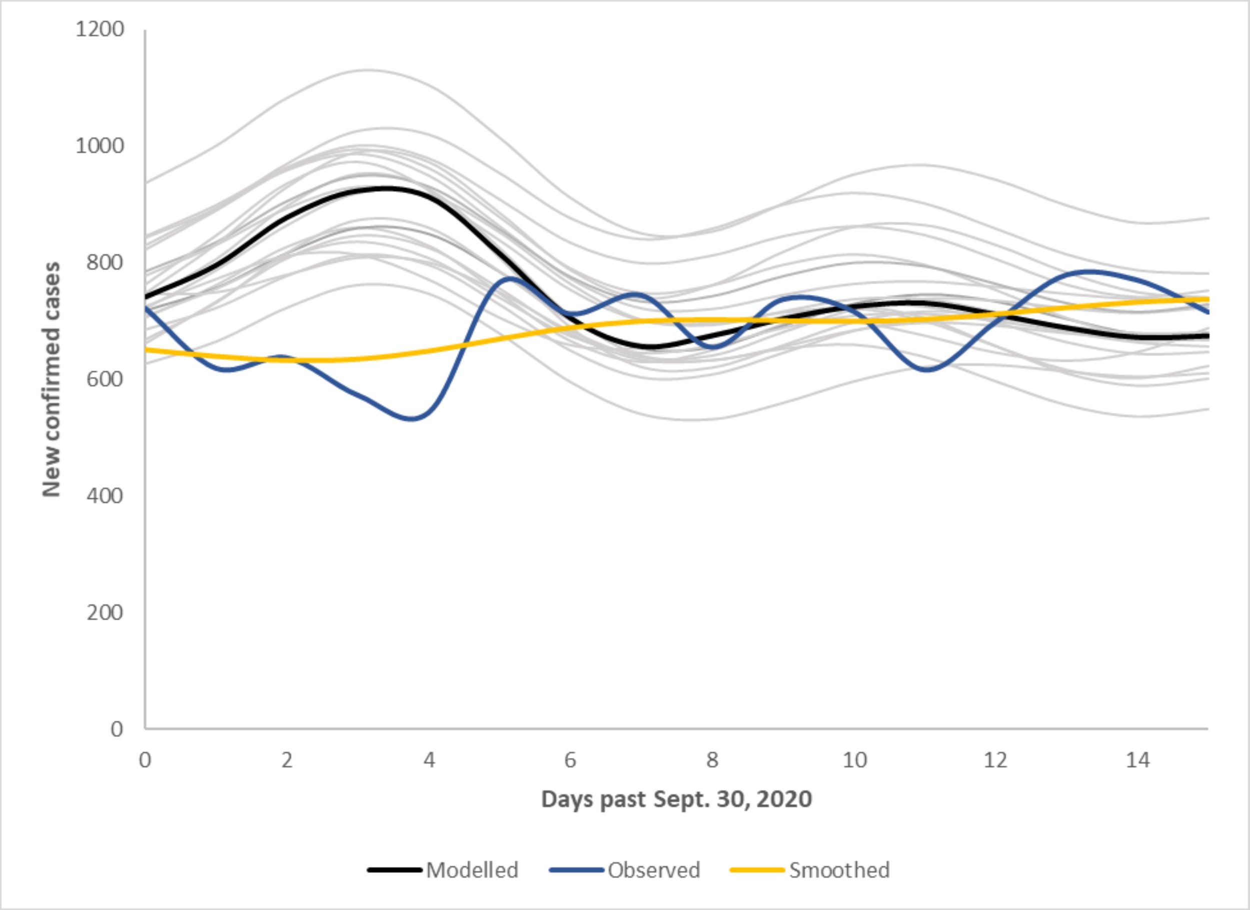


Observed cases in the CCM plus database (blue), after Gaussian kernel smoothing with a bandwidth of 2 days (gold), compared to the average among 20 model repetitions (black) and to the individual calibrated replicates (grey) along between September 1 and 30, 2020. Note, both observed and modelled counts subjected to Gaussian kernel smoothing with a bandwidth of 2 days.

© 2021 Naimark D et al. *JAMA Network Open.*

**eFigure 14.** Distribution of Numbers of Day Care, Primary, Elementary and High School Classroom Closures for Scenarios in Which Schools Had Reopened

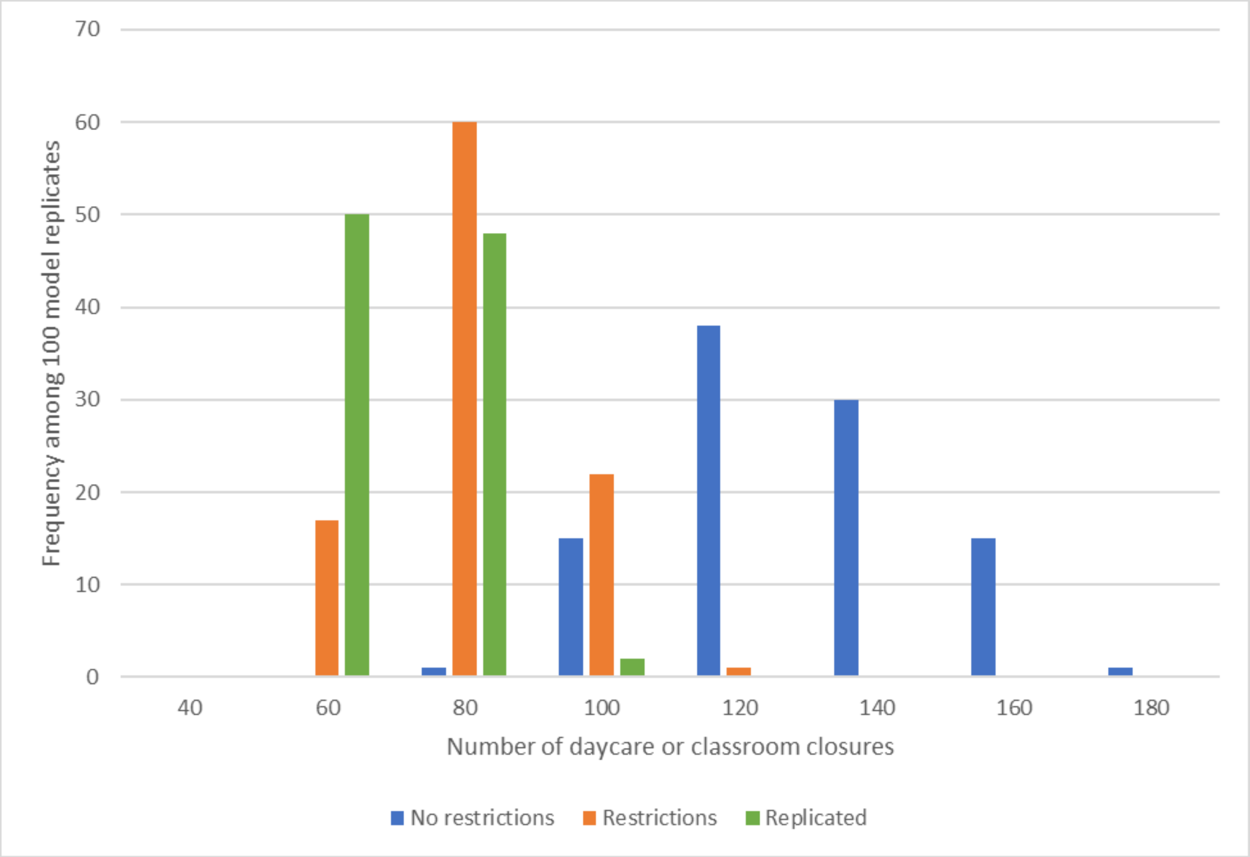


Distributions of the estimated numbers of classroom closures on model scale among 100 simulation model replicates. Scenario 1b - for the case that no restrictions were implemented (blue); scenario 2b – for the case that restrictions were implemented (orange); scenario 3b - the slowing of the growth of new case numbers observed from October 1 – 15, 2020, had persisted until October 31, 2020 (green).

© 2021 Naimark D et al. *JAMA Network Open.*

**eFigure 15.** Distribution of the Percentage of SARS-CoV-2 Infections Acquired in Schools for Students and Teachers in Scenarios in Which Schools Had Reopened

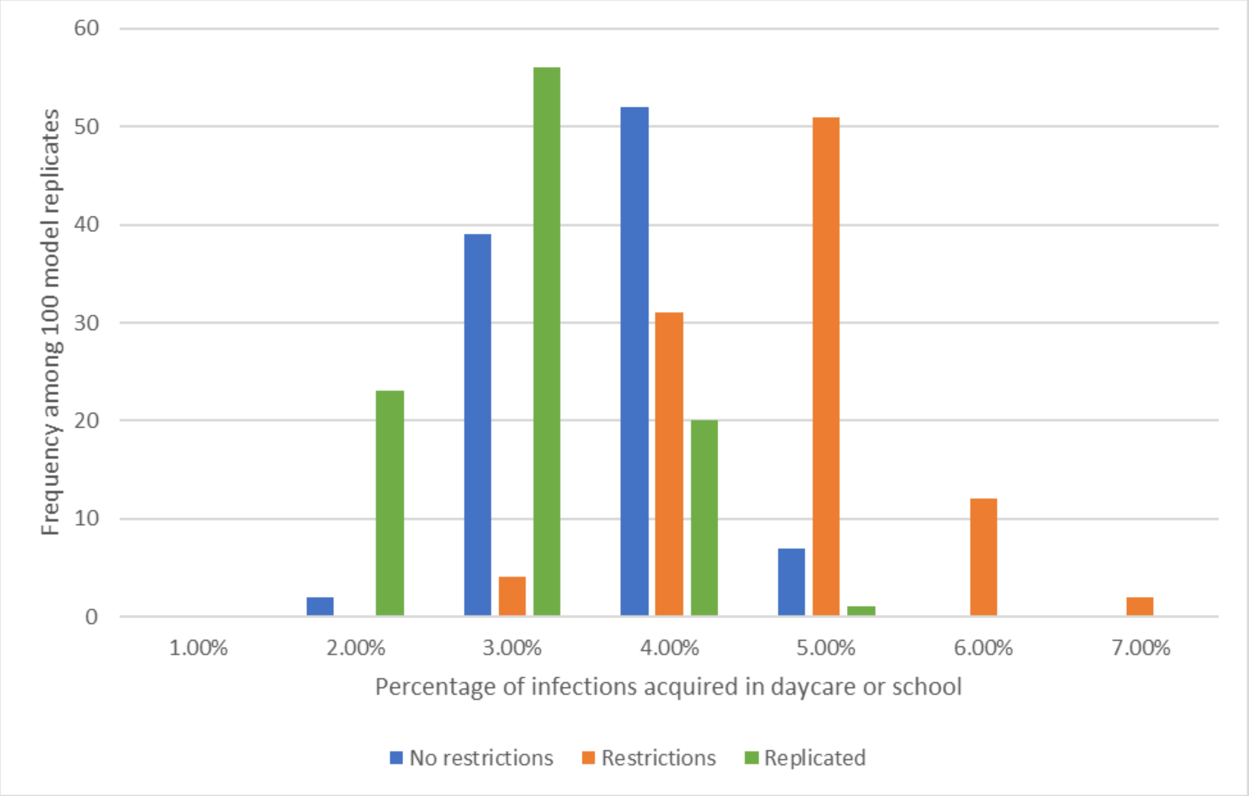


Distributions of the estimated percentage of COVID-19 infections acquired in school among the total number of infections among students and teachers on model scale among 100 simulation model replicates. Scenario 1b - for the case that no restrictions were implemented (blue); scenario 2b – for the case that restrictions were implemented (orange); scenario 3b - the slowing of the growth of new case numbers observed from October 1 – 15, 2020, had persisted until October 31, 2020 (green).

© 2021 Naimark D et al. *JAMA Network Open.*

**eFigure 16.** Model Simulation Results for Scenario 1A

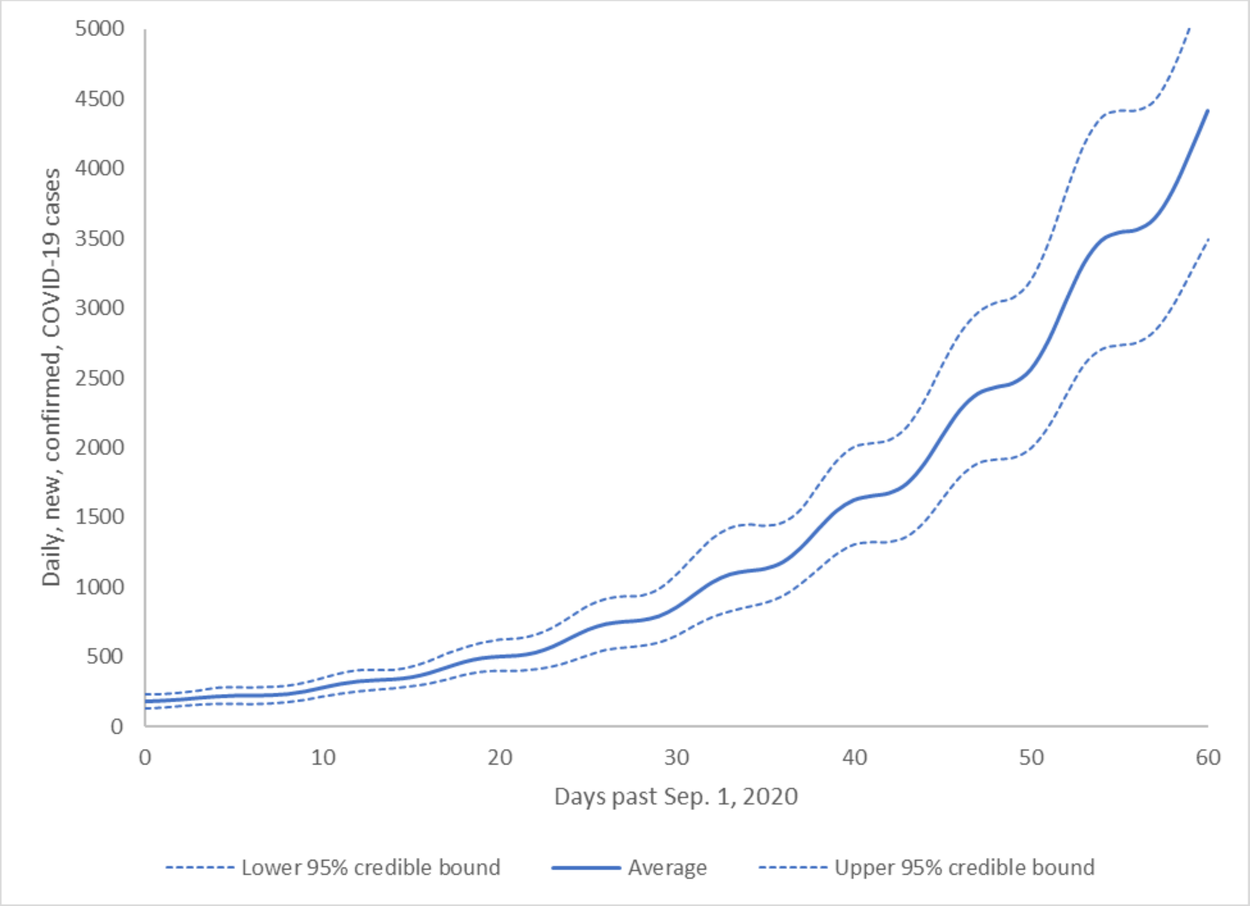


No nonpharmaceutical interventions (NPIs) restricting contacts and/or reducing the probability of transmission of COVID-19 between contacts had been implemented (that is, the trends in the rise of new cases in September *had been allowed* to persist through October) and schools *remained closed* on September 15, 2020. Average of 100 model replicates (solid blue) along with the associated 2.5$^{th}$ and 97.5$^{th}$ percentiles (dotted blue) between September 1 and October 31, 2020. Note, modelled counts subjected to Gaussian kernel smoothing with a bandwidth of 2 days.

© 2021 Naimark D et al. *JAMA Network Open.*

**eFigure 17.** Model Simulation Results for Scenario 1B

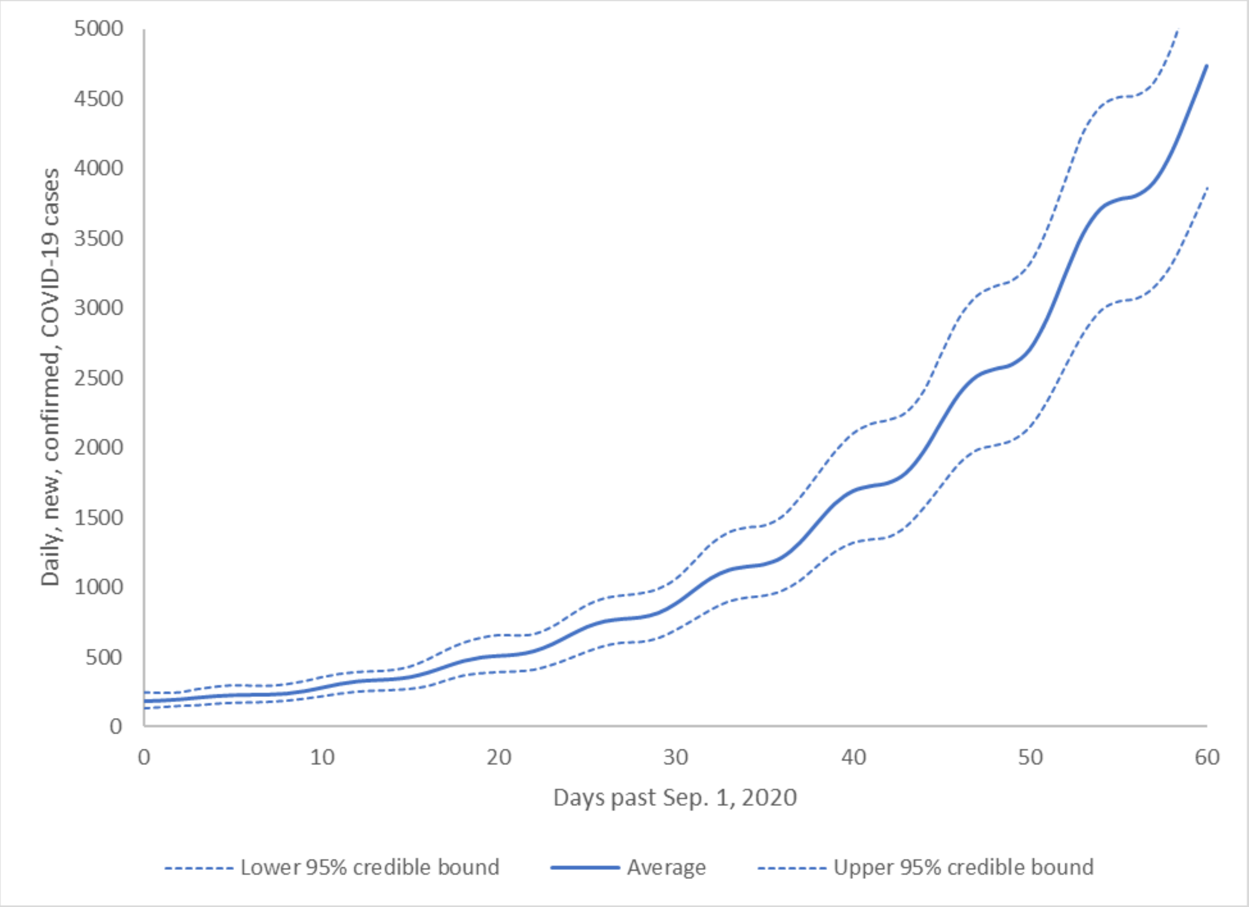


No nonpharmaceutical interventions (NPIs) restricting contacts and/or reducing the probability of transmission of COVID-19 between contacts had been implemented (that is, if the trends in the rise of new cases in September *had been allowed* to persist through October) and schools *had re-opened* on September 15, 2020. Average of 100 model replicates (solid blue) along with the associated 2.5$^{th}$ and 97.5$^{th}$ percentiles (dotted blue) between September 1 and October 31, 2020. Note, modelled counts subjected to Gaussian kernel smoothing with a bandwidth of 2 days.

© 2021 Naimark D et al. *JAMA Network Open.*

**eFigure 18.** Model Simulation Results for Scenario 2A

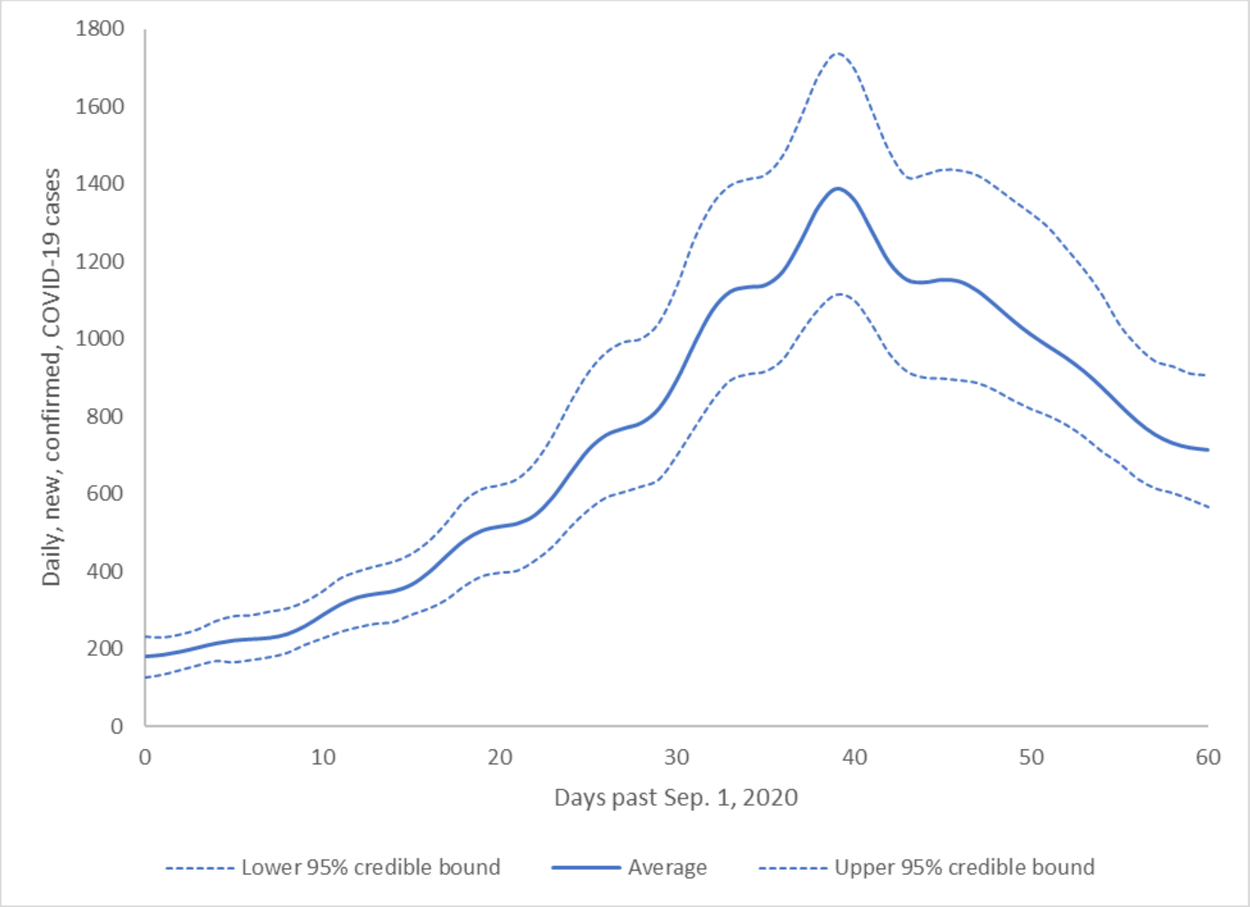


Nonpharmaceutical interventions (NPIs) restricting contacts and/or reducing the probability of transmission of COVID-19 between contacts had been implemented on October 1, 2020 (that is, if the trends in the rise of new cases in September *had not been allowed* to persist through October) and schools *remained closed* on September 15, 2020. Average of 100 model replicates (solid blue) along with the associated 2.5th and 97.5th percentiles (dotted blue) between September 1 and October 31, 2020. Note, counts subjected to Gaussian kernel smoothing with a bandwidth of 2 days.

© 2021 Naimark D et al. *JAMA Network Open.*

**eFigure 19.** Model Simulation Results for Scenario 2B

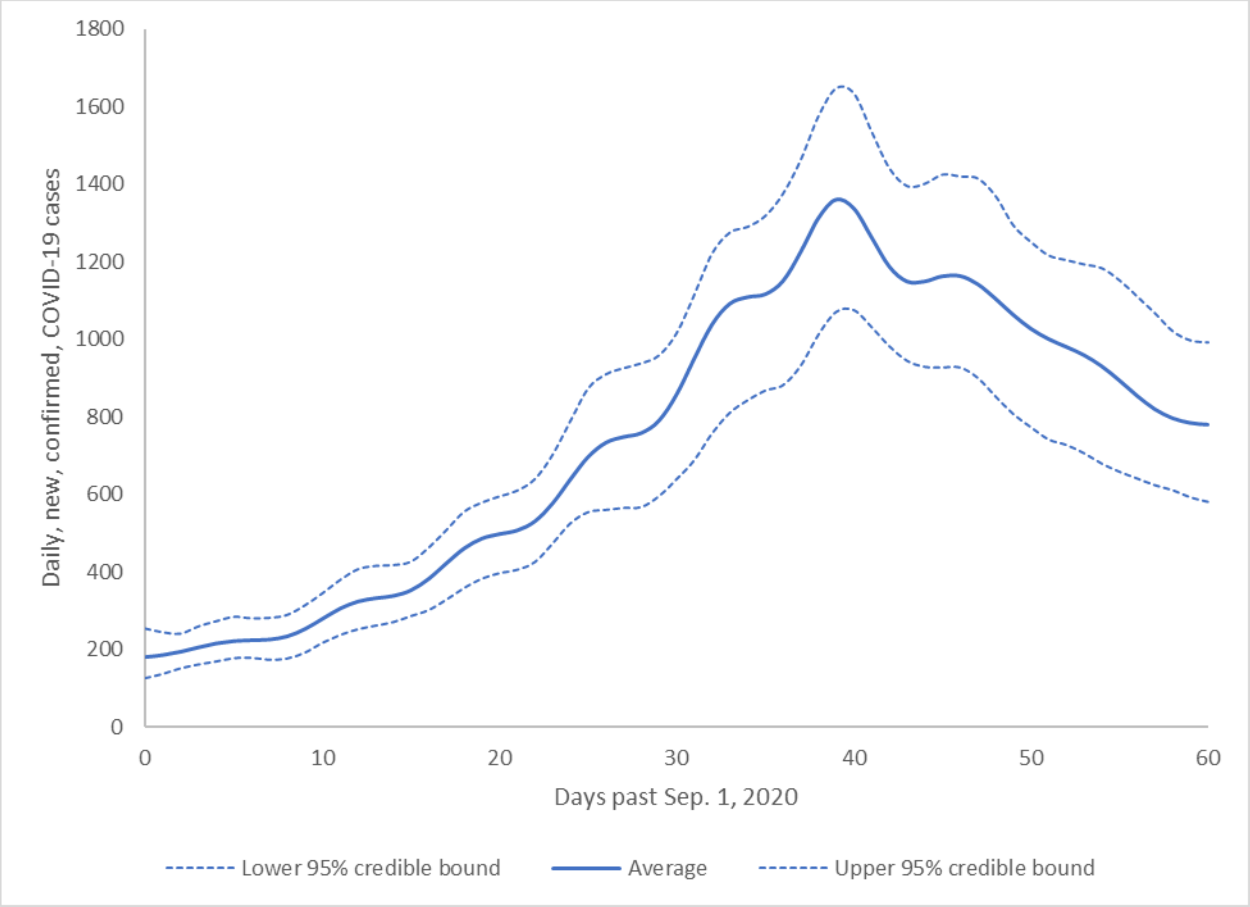


Nonpharmaceutical interventions (NPIs) restricting contacts and/or reducing the probability of transmission of COVID-19 between contacts were implemented on October 1, 2020 (that is, if the trends in the rise of new cases in September *had not been allowed* to persist through October) and schools *had re-opened* on September 15, 2020. Average of 100 model replicates (solid blue) along with the associated 2.5$^{th}$ and 97.5$^{th}$ percentiles (dotted blue) between September 1 and October 31, 2020. Note, counts subjected to Gaussian kernel smoothing with a bandwidth of 2 days.

© 2021 Naimark D et al. *JAMA Network Open.*

**eFigure 20.** Model Simulation Results for Scenario 3A

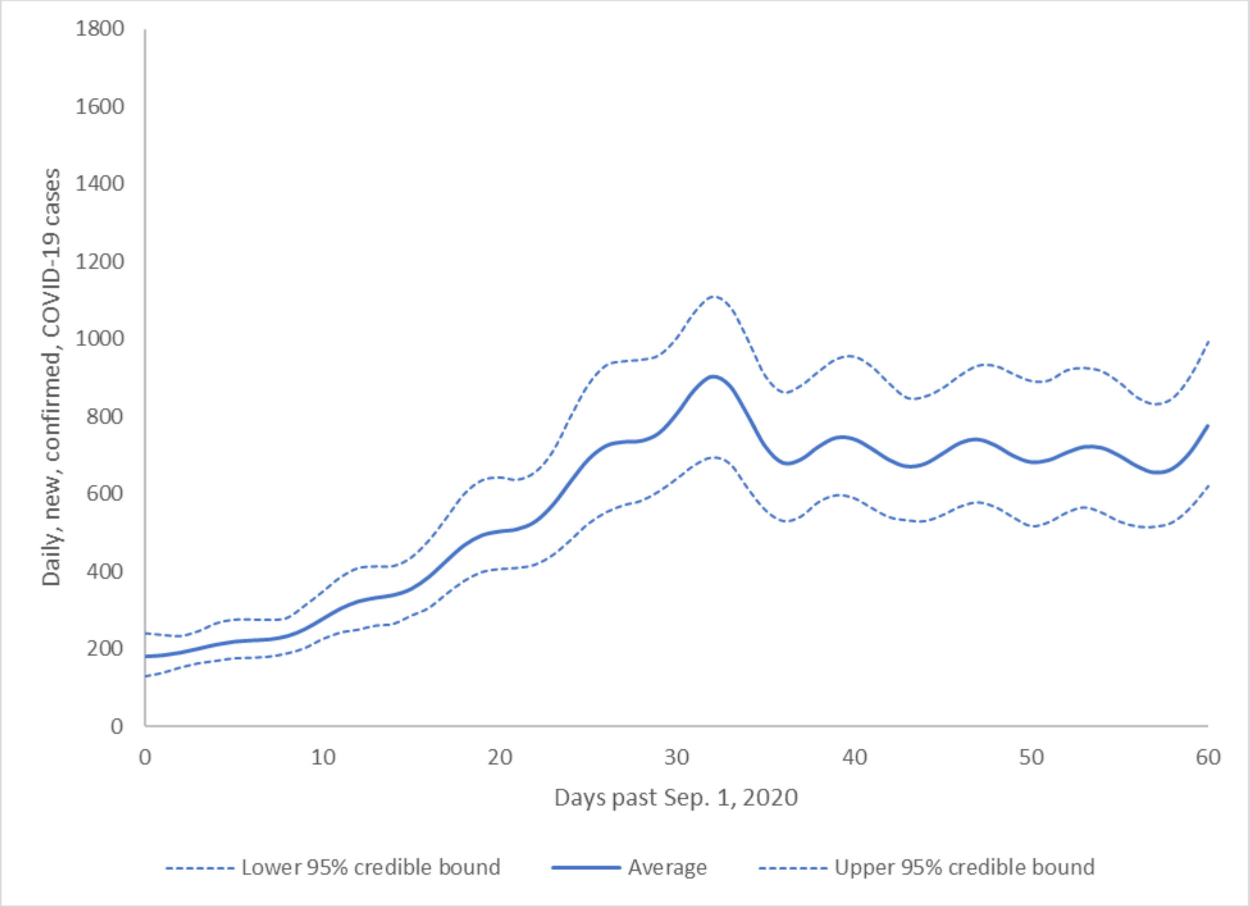


The reduction of the growth in new, daily, confirmed, COVID-19 cases had reduced to that observed between October 1 – 15, 2020, the lower growth rate had persisted until Oct 31, 2020, and schools *remained closed* on September 15, 2020. Average of 100 model replicates (solid blue) along with the associated 2.5$^{th}$ and 97.5$^{th}$ percentiles (dotted blue) between September 1 and October 31, 2020. Note, counts subjected to Gaussian kernel smoothing with a bandwidth of 2 days.

© 2021 Naimark D et al. *JAMA Network Open.*

**eFigure 21.** Model Simulation Results for Scenario 3B

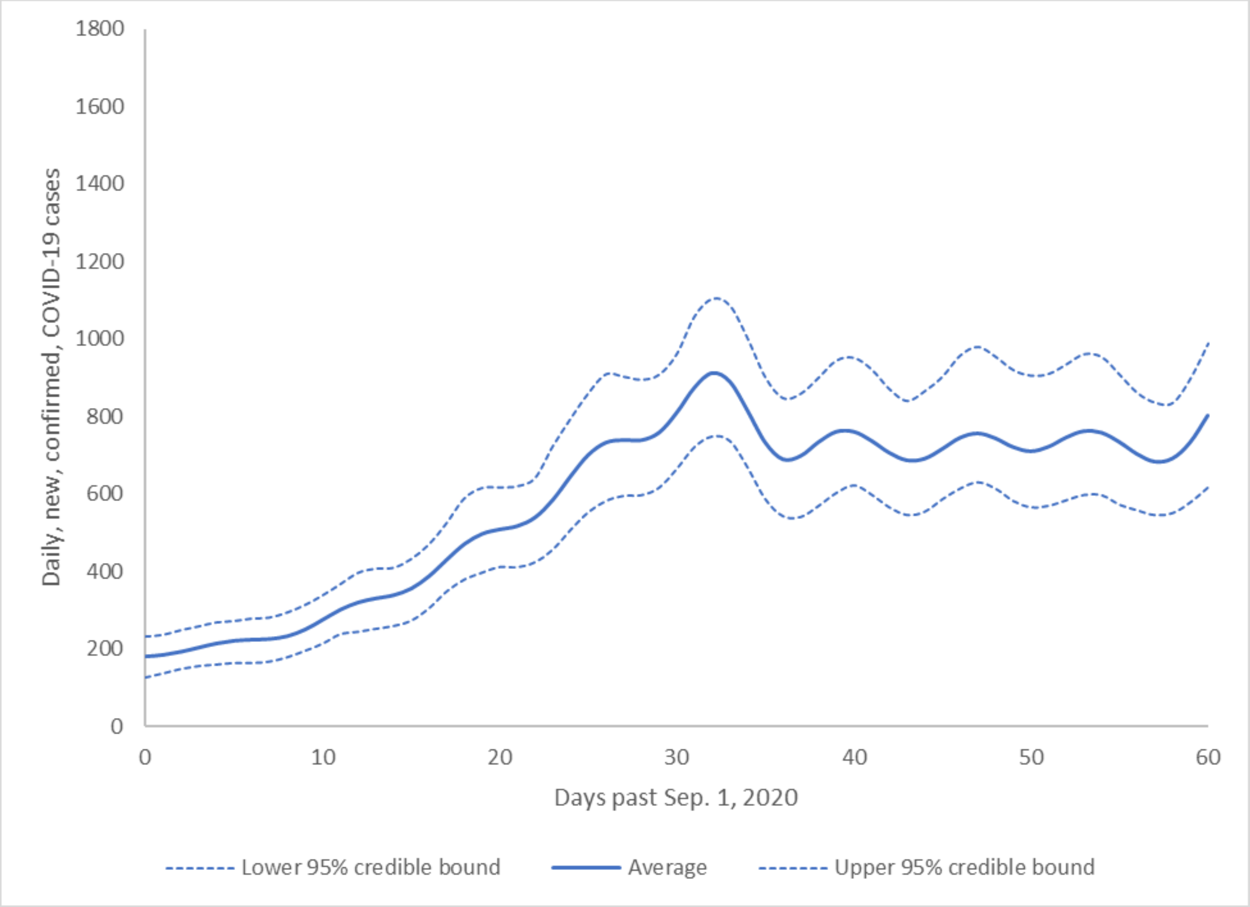


The reduction of the growth in new, daily, confirmed, COVID-19 cases had reduced to that observed between October 1 – 15, 2020, that the lower growth rate had persisted until Oct 31, 2020, and schools *had re-opened* on September 15, 2020. Average of 100 model replicates (solid blue) along with the associated 2.5$^{th}$ and 97.5$^{th}$ percentiles (dotted blue) between September 1 and October 31, 2020. Note, counts subjected to Gaussian kernel smoothing with a bandwidth of 2 days.

© 2021 Naimark D et al. *JAMA Network Open.*

**eFigure 22.** Reduction of Cumulative Cases Between September 1 and October 31, 2020, Attributable to the 2 Policy Choices to Implement Public Health Restrictions or Not vs to Open Schools or Not

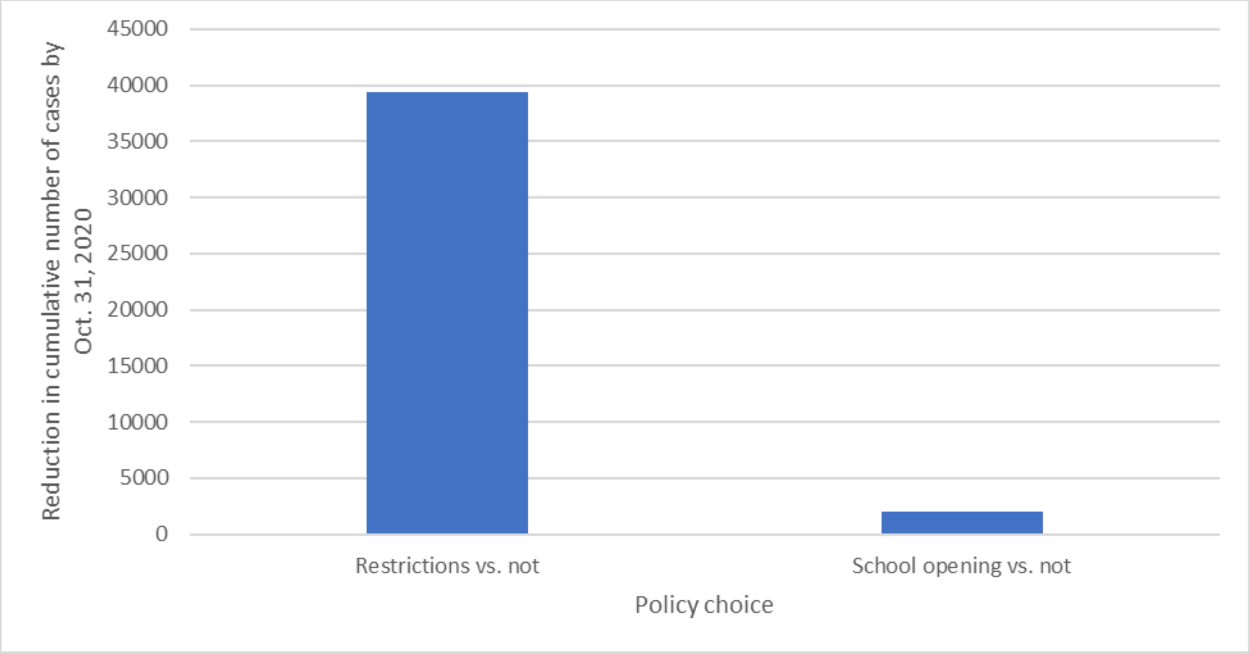


Estimated reduction of cumulative COVID-19 cases between September 1 and October 31, 2020, attributable to the two policy choices: to implement nonpharmaceutical interventions (NPIs) or not (the mean of the estimated cumulative cases for scenarios 1a and 1b minus the mean estimates for scenarios 2a and 2b) vs. to open schools or not (the mean estimates for scenarios 1b and 2b minus the mean estimates for scenarios 1a and 2a).

© 2021 Naimark D et al. *JAMA Network Open.*

**eFigure 23.** Sensitivity Analysis for Effectiveness of School-Based Mitigation of SARS-CoV-2 Transmission

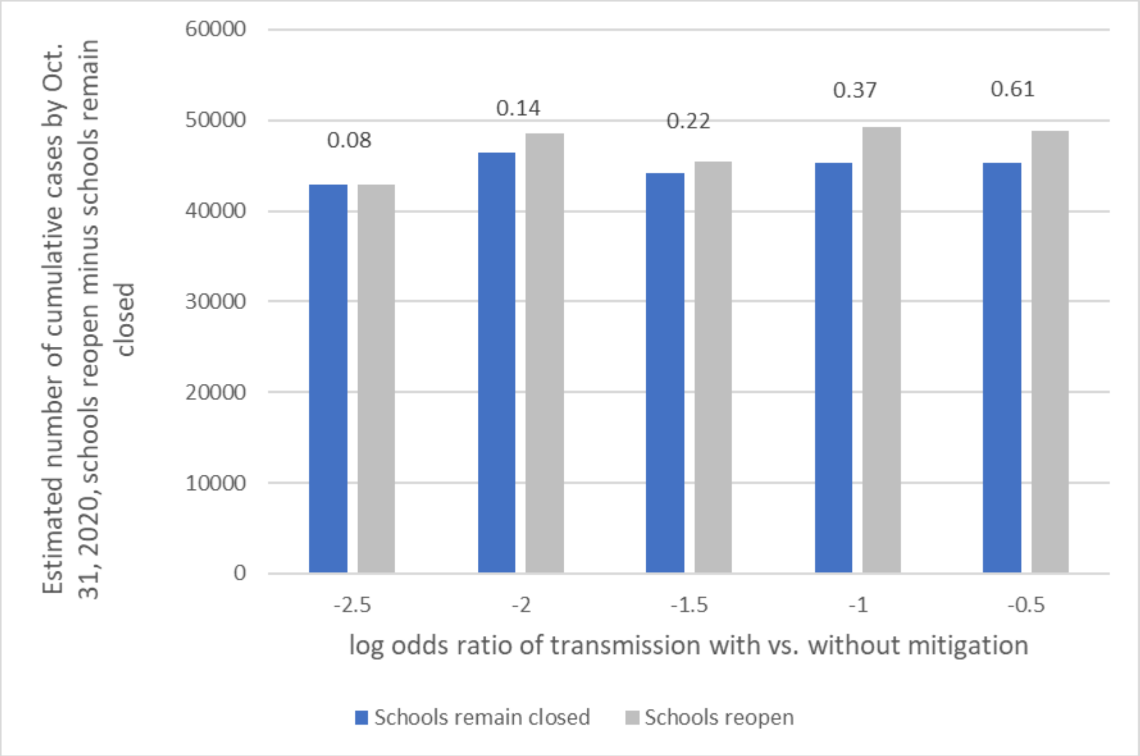


The difference between school opening on September 15, 2020, versus remaining closed, in the estimated mean cumulative case numbers on October 31, 2020, across 100 model repetitions is shown for various degrees of effectiveness of measures to mitigate transmission of COVID-19 among children at school as indicated in the main text. The measure of effectiveness is the log odds ratio for transmission with versus without mitigation. The corresponding odds ratio is shown as labels above each pair of columns in the figure. Reduction of contact numbers and the probability of transmission per contact were held at their base case values for non-pharmaceutical interventions being imposed on October 1, 2020 (Table 1 in the main text).

**eFigure 24.** Sensitivity Analysis for Effectiveness of Community-Based, Nonpharmaceutical Interventions

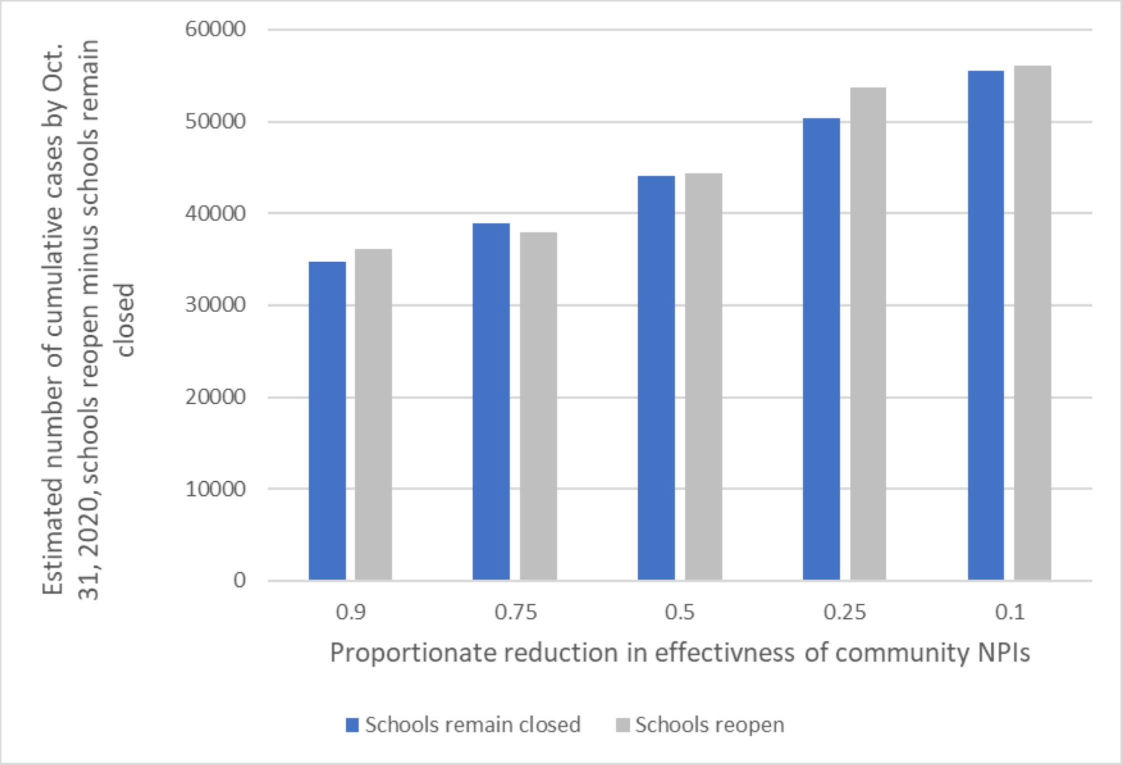


The difference between school opening on September 15, 2020, versus remaining closed in the estimated mean cumulative case numbers on October 31, 2020, across 100 model repetitions is shown for various degrees of the proportionate reduction in both contact numbers and the probability of transmission per contact after the introduction of community-based non-pharmaceutical interventions on October 1, 2020, relative to pre-pandemic values and prior to imposition of community-based NPIs. The odds ratio of transmission for school mitigation versus no mitigation was held at its base case value (Table 1 in the main text).

© 2021 Naimark D et al. *JAMA Network Open.*

**eReferences**


1. Statistics Canada. Social Policy Simulation Database and Model (SPSD/M) Version 28.0.1. 2020. https://search2.odesi.ca/#/details?uri=%2Fodesi%2Fspsd-89F0002-E.xml.
2. Statistics Canada. *Rural and Small Town Canada Analysis Bulletin (Cat.21-006-XIE)*.; 2001.
3. Ontario Ministry of Health. *Integrated Public Health Information Systems (IPHIS)*.; 2020.
4. Tuite AR, Fisman DN, Greer AL. Mathematical modelling of COVID-19 transmission and mitigation strategies in the population of Ontario, Canada. *Cmaj*. 2020;192(19):E497-E505. doi:10.1503/cmaj.200476
5. Ontario Public Health. *COVID-19 Seroprevalence in Ontario: March 27, 2020 to June 30, 2020*.; 2020.
6. Brisson, Marc; Gingras, Guillaume; Drolet, Melanie; Laprise J-F. Modélisation de l'évolution de la COVID-19 au Québec. http://www.marc-brisson.net/covid19-response/Epidemiologie-et-modelisation-evolution-COVID-19-au-Quebec_16-octobre-2020.pdf.
7. Statistics Canada. *Percentage of Workforce Teleworking or Working Remotely, and Percentage of Workforce Expected to Continue Teleworking or Working Remotely after the Pandemic, by Business Characteristics*.
8. Powell M. *The BOBYQA Algorithm for Bound Constrained Optimization without Derivatives*. Cambridge; 2010. http://www.optimization-online.org/DB_HTML/2010/05/2616.html.
9. Wang, S; Kustra, R; Watts, A; Khan K; Mishra S. *GTA Proxy Measures of Physical Distancing*.; 2020.


© 2021 Naimark D et al. *JAMA Network Open.*
